# Genomic Insights Into the Acid Adaptation of Novel Methanotrophs Enriched From Acidic Forest Soils

**DOI:** 10.3389/fmicb.2018.01982

**Published:** 2018-08-27

**Authors:** Ngoc-Loi Nguyen, Woon-Jong Yu, Joo-Han Gwak, So-Jeong Kim, Soo-Je Park, Craig W. Herbold, Jong-Geol Kim, Man-Young Jung, Sung-Keun Rhee

**Affiliations:** ^1^Department of Microbiology, Chungbuk National University, Cheongju, South Korea; ^2^Geologic Environment Research Division, Korea Institute of Geoscience and Mineral Resources, Daejeon, South Korea; ^3^Department of Biology, Jeju National University, Jeju City, South Korea; ^4^Department of Microbiology and Ecosystem Science, Division of Microbial Ecology, University of Vienna, Vienna, Austria

**Keywords:** methanotroph, acid adaptation, genome reconstruction, comparative genomics, *Methylocystis*, *Methylobacter*

## Abstract

Soil acidification is accelerated by anthropogenic and agricultural activities, which could significantly affect global methane cycles. However, detailed knowledge of the genomic properties of methanotrophs adapted to acidic soils remains scarce. Using metagenomic approaches, we analyzed methane-utilizing communities enriched from acidic forest soils with pH 3 and 4, and recovered near-complete genomes of proteobacterial methanotrophs. Novel methanotroph genomes designated KS32 and KS41, belonging to two representative clades of methanotrophs (*Methylocystis* of *Alphaproteobacteria* and *Methylobacter* of *Gammaproteobacteria*), were dominant. Comparative genomic analysis revealed diverse systems of membrane transporters for ensuring pH homeostasis and defense against toxic chemicals. Various potassium transporter systems, sodium/proton antiporters, and two copies of proton-translocating F1F0-type ATP synthase genes were identified, which might participate in the key pH homeostasis mechanisms in KS32. In addition, the V-type ATP synthase and urea assimilation genes might be used for pH homeostasis in KS41. Genes involved in the modification of membranes by incorporation of cyclopropane fatty acids and hopanoid lipids might be used for reducing proton influx into cells. The two methanotroph genomes possess genes for elaborate heavy metal efflux pumping systems, possibly owing to increased heavy metal toxicity in acidic conditions. Phylogenies of key genes involved in acid adaptation, methane oxidation, and antiviral defense in KS41 were incongruent with that of 16S rRNA. Thus, the detailed analysis of the genome sequences provides new insights into the ecology of methanotrophs responding to soil acidification.

## Introduction

Soil methanotrophs play a significant role globally in the biogeochemical cycling of methane (Le Mer and Roger, 2001; Dutaur and Verchot, 2007) by oxidizing atmospheric methane and oxidizing subsurface methane before it can be released to the atmosphere (Semrau, 2011; Knief, 2015; Strong et al., 2015). Methanotrophs are group bacteria that utilize methane as their sole carbon and energy source (Söhngen, 1906; Hanson and Hanson, 1996; Raghoebarsing et al., 2006). Typical aerobic terrestrial methanotrophs belong to the classes *Alphaproteobacteria* (Type II) and *Gammaproteobacteria* (Type I). Methanotrophic bacteria belonging to the phylum *Verrucomicrobia* (Dunfield et al., 2007) and candidate phylum NC10 (Ettwig et al., 2010; He et al., 2016) have been reported from acidic thermal environments and anoxic environments, respectively. The key step in aerobic methane oxidation to methanol is catalyzed by the methane monooxygenase which occurs as a particulate, membrane bound form (pMMO) and as a soluble, cytosolic form (sMMO).

Soil acidification is accelerated by anthropogenic and agricultural activities, and naturally acidic soils (pH ≤ 5) comprise about 30% of soils in the world (Behera and Shukla, 2015; Yu et al., 2015; Goulding, 2016). Upland forest soils have had less attention in terms of methane production and emission studies although those cover larger areas than wetlands (Megonigal and Guenther, 2008; Mukhin and Voronin, 2011). The upland forest soils harbor populations of methanogens. Thus, the upland forest soils can become net sources of methane especially when water content increases (Megonigal and Guenther, 2008; Hofmann et al., 2016). Since pH values of pine-dominated forest soils typically range between 3 and 4 (Lau et al., 2007; Megonigal and Guenther, 2008; Machacova et al., 2016), which is below the pH optimum of many cultured methanotrophs (Hanson and Hanson, 1996), acid adaptation of methanotrophs needs to be adequately characterized to evaluate methane oxidation in upland forest ecosystems.

Methanotrophic communities can be influenced by pH through (1) the effect of pH on any associated microbial communities and (2) by changing the availability of toxic elements and nutrients (Kizilova et al., 2013; Zhou et al., 2017). Methane oxidation activity in natural forest soils was shown to be suppressed under acidic conditions or was tolerant to this effect (Amaral et al., 1998; Saari et al., 2004). However, detailed genomic knowledge concerning the response of methanotrophs to acidification of soil remains scarce (Aronson et al., 2013).

Recent studies have identified diverse methanotrophs including novel uncultured clades in acidic environments (Kip et al., 2011; Arai et al., 2014; Baesman et al., 2015). Acidophilic methanotrophs of the alphaproteobacterial families *Methylocystaceae* and *Beijerinckiaceae* are common inhabitants of acidic environments (Dedysh, 2011). Two moderately acidophilic methanotrophs, *Methylocystis heyeri* and *Methylocystis bryophila*, in the family *Methylocystaceae* were isolated from acidic *Sphagnum* peat and have an optimal growth at pH 5.8 and 6.5, respectively (Dedysh et al., 2007; Belova et al., 2013). Methanotrophs from three genera (*Methylocella, Methyloferula*, and *Methylocapsa*), belonging to the family *Beijerinckiaceae*, have been isolated from acidic habitats such as *Sphagnum* peat bogs and acidic forest soils. Members of these genera have a growth optimum at pH 4.8–5.5 (Dedysh et al., 2000, 2002, 2004; Vorobev et al., 2011). Acidophilic methanotrophs from the gammaproteobacterial family *Methylococcaceae* have also been isolated from various acidic environment. These include the acidophilic *Methylomonas* sp. M5 and *Methylovulum* sp. M200 (Kip et al., 2011); acid-tolerant species *Methylomonas paludis* and *Methylovulum psychrotolerans*, with the ability to grow at pH as low as 4.0 (Danilova et al., 2013; Oshkin et al., 2016); and thermophilic strains *Methylocaldum* sp. BFH1 and BFH2 with optimal pH 5.5–6.0, respectively (Islam et al., 2016). Although members of *Methylobacter* have been detected in acidic forest soils, *Sphagnum* peatlands, and acidic enrichment cultures (Kolb et al., 2005; Kip et al., 2011; Baesman et al., 2015), no taxonomically characterized isolate belonging to the *Methylobacter* has shown capacity to grow at a pH below 5.0, including the recently descried taxa *Methylobacter tundripaludum* (Bowman et al., 1993; Wartiainen et al., 2006).

Comparative genomic analysis provides insights into metabolic pathways and eco-physiological properties (Kelly et al., 2005; Dam et al., 2013; Tamas et al., 2014; Vorobev et al., 2014). A previous genomic study of an extremely acidophilic verrucomicrobial methanotroph identified several candidate genes that may be important for its acidophilic lifestyle (Hou et al., 2008). However, only a limited number of genome sequences of acidophilic and acid-tolerant methanotrophs are available, particularly for Type I gammaproteobacterial methanotrophs. Studies on the comparative genomics of acidophilic proteobacteria are rare, further limiting our understanding of the physiological and biochemical mechanisms which support methanotrophic life under acidic conditions.

Here, we characterized methanotroph communities enriched from acidic forest soils using sequencing batch reactors (SBRs) at pH 3 and 4. Using a metagenomic approach, we reconstructed genomes for two dominant methanotrophs belonging to the genera *Methylocystis* and *Methylobacter*. This study will help to expand our knowledge of the physiology and ecology of methanotrophs in acidic soils.

## Materials and Methods

### Enrichment of Methanotrophs From Acidic Soils

Forest soil samples were collected from Korean pine (*Pinus koraiensis*)-dominated forests, but also having other tree species such as *Quercus mongolica* and *Abies holophylla* at Chungbuk National University and Gutdae Mountain sites at Cheongju, South Korea. The forest soil samples were consisted of the upper layers of organic matter (0–5 cm, pH 4.5–5.3) and the lower layers of mineral material mixed with organic matter (5–10 cm, pH 4.0). The soil samples from lower layer were collected and transported to the laboratory and used for inoculation. The locations and general properties of two soils are presented in **Supplementary Table S1**. The soils were frozen at -80°C before extraction of DNA using the soil prep kit (GeneAll, Korea). Quantification of bacterial 16S RNA gene, *pmoA*, and *mmoX* copy numbers in the soils was carried out using CFX Connect^TM^ Real-Time System (Bio-Rad Laboratories, Hercules, CA, United States) and built in CFX manager software (version 3.0, Bio-Rad Laboratories, Hercules, CA, United States). The DNA in the range of 10–20 ng was used for quantification of target gene copies with the following primers: 518F/786R for 16S rRNA gene (Muyzer et al., 1993), A189F/mb661R for *pmoA* (Kolb et al., 2003), and 536F/898R for *mmoX* (Fuse et al., 1998). Standard curves generated for each run by using reference gene standards with gene copies ranging from 0 to 10^9^ per reaction were used to estimate gene copy numbers in samples.

Two methanotrophic communities, designated as R4 and R3, were enriched from a mixture of the two soils in SBRs at pH 4 and 3, respectively, to increase diversity of methanotrophs by retaining slow-growing methanotrophs. The volume of each reactor was 5 L, consisting of 3 L of medium and 2 L of headspace with methane (20% v/v in headspace). Two reactors were fitted with a fermenter lid containing a stirrer, a dissolved oxygen probe, a pH probe, acid and base in-flow tubes for pH control, temperature detector, gas line, feed in-flow tube, sampling line, and effluent-withdraw line. The low-salt mineral (LSM) medium (0.4 mM MgSO_4_⋅2H_2_O, 0.1 mM CaCl_2_⋅2H_2_O, 0.2 mM K_2_SO_4_, and 1 mM KH_2_PO_4_) was supplemented with final concentrations of 1 mL/L trace element solution, 100 μL/L vitamin solution (Widdel and Bak, 1992), and 0.2 μM cerium chloride. NH_4_Cl (0.1 mM) was supplied as a nitrogen source. pH was adjusted to pH 4 and 3 with 0.2 N H_2_SO_4_, for enrichments of R4 and R3, respectively. Each reactor was inoculated with soil (200 g/L each) and was operated at 30 ± 1°C, with stirring at 500 rpm. The reactors were operated as SBRs with a cycle of 2 weeks, comprised of three phases: (i) settling period for 12 h, (ii) withdrawal period for 0.5 h, during which 75% of the effluent was replaced with an equal volume of fresh LSM, and the methane/air was replaced at the same ratio of 1:4 in the headspace, (iii) and reaction period in which biomass was incubated for about 13.5 days with continuous stirring. Reactor performance was checked using subsamples of the SBR biomass. In brief, 25 mL sub-cultures were taken from the SBRs and incubated in 150 mL-serum vials with methane (20% v/v) in headspace in a shaking incubator. From the headspace, gas samples (100 μL) were taken and methane consumption was measured using gas chromatography GC-2010 Plus (SHIMADZU, Japan). A GC/FID was equipped with a Rtx-1 GC column (film thickness, 0.25 μm; inside diameter, 0.25 mm; length, 30 m; Restek, Bellefonte, PA, United States) and a flame ionization detector. Nitrogen gas was used as a carrier, with a column flow rate of 1 mL/min. The chromatographic conditions: injector temperature, 150°C (split ratio of 1:10); oven temperature of 80°C held for detection time; detector temperature, 200°C.

After 3 months, biomass was harvested from 1 L of culture by centrifugation at 10,000 × *g* for 10 min. The pellet was placed in a sterile conical tube and stored at -80°C until further processing for community analysis based on PCR amplification of 16S rRNA gene. For differential coverage-based binning of the methanotroph genomes from the metagenomes of the enrichment cultures, 1% of the culture from SBR was transferred to a 2 L bottle containing 1 L of a fresh LSM and 1 L of headspace with methane (10% v/v headspace) and incubated at 25°C. After depletion of methane, the biomass was harvested for extraction of metagenomic DNA for sequencing.

### Whole DNA Extraction and Sequencing

Total DNA was extracted from each pellet using a modified CTAB bead-beating method (Griffiths et al., 2000) to obtain metagenomic DNA extracts (R3 and R4). Briefly, biomass was ground with quartz and liquid nitrogen and treated with DNA extraction buffer at 65°C for 15 min, and the nucleic acids were purified with chloroform/isoamyl alcohol. Metagenomic DNA integrity was confirmed using 0.8% (w/v) agarose gel electrophoresis and DNA was quantified using a NanoDrop ND-1000 spectrophotometer (Thermo Fisher Scientific, Waltham, MA, United States).

For community analysis, the V4 and V5 region of the 16S rRNA gene were amplified using primers 515F (5′-GTG YCA GCM GCC GCG GTA A-3′) and 926R (5′-CCG YCA ATT YMT TTR AGT TT-3′) (Osburn et al., 2011), with sample indexing adapters (Nextera XT index kit). PCR amplifications were conducted via the following steps: 5 min heating step at 95°C, followed by 25 cycles at 95°C for 45 s (denaturation), 50°C for 45 s (annealing), 68°C for 90 s (extension), and a final extension at 72°C for 5 min. Sequencing of 300 bp paired-end reads on the Illumina MiSeq platform was performed by Macrogen Inc., South Korea. The two metagenomic DNA extracts from the cultures derived from R3 and R4 were sequenced on the Illumina Hiseq 2000 (Illumina, San Diego, CA, United States) and Pacbio RSII platforms (Pacific Biosciences, Menlo Park, CA, United States) by Macrogen Inc., South Korea.

### 16S rRNA Gene Amplicon Analyses

Reads were examined using FastQC v0.11.3, quality trimming and adapter removal were carried out with Trimmomatic v0.36 with default settings. Qualified paired-end reads were merged using the commercial software Geneious (version 9.1.8). For classification, merged reads were analyzed from SILVAngs web server (v. 1.7.0^1^) (Andreas et al., 2014). The diversity and species richness indices were calculated by alpha_diversity.py script in Qiime (Caporaso et al., 2010) with rarified counts.

### Metagenome Assembly and Annotation

Illumina data were quality trimmed with Sickle software (Joshi and Fass, 2011) and pooled with Pac-Bio reads, followed by hybrid-assembly using SPAdes v3.5.0 (Bankevich et al., 2012). Coding sequences (CDSs) in the assembled data were predicted using Prodigal in metagenomic mode (Hyatt et al., 2010). rRNAs and tRNAs were identified using RNAmmer (Lagesen et al., 2007) and tRNAscan-SE (Lowe and Chan, 2016), respectively. Functional predictions of the protein sequences were performed using BLASTp similarity searches based on the best BLAST hit against NCBI NR database (best bit score, cut off: query shared >80% similarity, >80% alignment coverage, and *e*-value 1*e^-^*^5^) and KEGG (GhostKOALA) (Kanehisa et al., 2017). Clusters of orthologous groups (COGs) (Tatusov et al., 2001) and TIGRfam (Haft et al., 2001) were assigned to predicted genes with rps-blast (*e*-value 1*e^-^*^5^). Domain information was obtained using the Pfam database.

### Genome Reconstruction and Comparative Genomics

Genomes of dominant microorganisms were reconstructed using differential coverage and tetranucleotide frequency as previously described (Albertsen et al., 2013). Trimmed HiSeq reads were mapped to the combined assembly with Bowtie2 (Langmead and Salzberg, 2012) and SAMtools (Li et al., 2009) to calculate coverage. CheckM v1.0.9 software was used to evaluate genomes (Parks et al., 2015) using criteria (completeness and contamination) to choose final bins for further analysis. Furthermore, all selected scaffolds were manually curated by taxonomic assignment of genes based on BLASTp results against the NR database (phylum level of hit gene to NR database; see above). Read depth of each scaffold, containing rRNA and *pmo/pxm* operons, was visualized using the R/Bioconductor package “Sushi” (Phanstiel et al., 2014). To check physical linkage of *pmoCAB* of KS41, PCR amplification of *pmoCAB* of KS41 in metagenomics DNA was conducted using following primers: K1-F (5′-CAGTGAAAGCTGATGCTGCG-3′) of *pmoC*, K2-R (5′-CGCTTCTGCACGAGACCTAA-3′) of *pmoA*, and K3-R (5′-ATCAGCAGTGCGACAAAGGA-3′) of *pmoB*. K1-F and K2-R pair and K1-F and K3-R pair were used for *pmoCA* (900 bp) and *pmoCAB* (1760 bp) amplification, respectively.

Genome bins were compared to reference genomes by calculating Orthologous Average Nucleotide Identity (OrthoANI) values and constructing phylogenomic trees with the Orthologous Average Identity Tool (Lee et al., 2015). Genome wide comparison and annotation of orthologous genes across multiple species were performed using OrthoVenn (Wang et al., 2015). The orthologous clusters were identified with default parameters, 1*e^-^*^5^ *e*-value cutoff for all protein similarity comparisons, and 1.5 inflation value for the generation of orthologous clusters. Clustered Regularly Interspaced Short Palindromic Repeats (CRISPRs) and specific families of tandem repeats were detected by the CRISPRFinder tool (Grissa et al., 2007).

### Phylogenetic Reconstructions

Single genes (16S rRNA gene and *pmoA* encoding for a subunit of the pMMO) were aligned using ClustalX (Thompson et al., 1997) and manually edited in BioEdit (Hall, 1999). Phylogenetic trees were constructed in MEGA7 (Kumar et al., 2016) using the Neighbor-Joining method (Saitou and Nei, 1987) on distances calculated with a Kimura 2-parameter model. Pairwise identity of 16S rRNA genes was determined with the Ezbiocloud server (Yoon et al., 2017). Phylogenetic analyses were also carried out for a set of 15 syntenic ribosomal proteins (Sorek et al., 2007; Castelle et al., 2015), pMMO proteins (PmoCAB), urea transporter proteins (UrtABCDF), and potassium transporter proteins (KdpFABCD). Derived protein sequences were aligned using ClustalX (Thompson et al., 1997) and sequence alignment data were joined using an online FASTA sequence toolbox, FaBox v. 1.41 (Villesen, 2007). Phylogenetic trees were constructed using the maximum-likelihood method under the best-fit model considering the relative rates of amino acid replacement in MEGA7. Bipartition confidence for all trees was assessed with 1000 bootstraps (Felsenstein, 1985).

## Results and Discussion

### Soil Properties and Enrichment Cultures

Soils used for obtaining enrichment cultures were acidic and typical for forest soils vegetated mostly by Korean pine (*P. koraiensis* L.). General properties are described in **Supplementary Table S1**. Methanotrophic communities were successfully enriched in SBRs at pH 4 and 3, which were designated R4 and R3, respectively. After biweekly refreshment of medium and headspace for 3 months, methane consumption was verified using 25 mL enrichment subsamples which consumed 0.5 mmol methane in 2 weeks (**Supplementary Figure S1**) with higher activities in the enrichments at pH 4 than at pH 3. The enrichment cultures obtained at the two different low pH values were used for comparison of the respective methanotroph communities.

### Methanotrophic Communities

**FIGURE 1:**
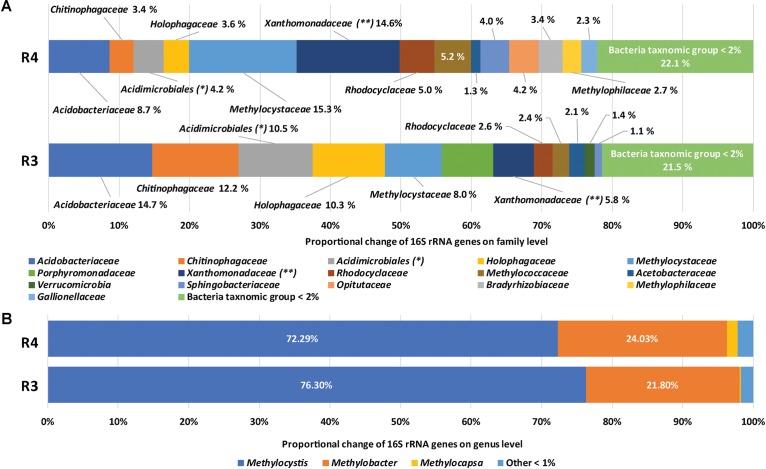
Taxonomic assignments of 16S rRNA gene sequences of methanotrophic enrichment cultures. Relative compositions of microbial communities **(A)** and methane-oxidizing bacteria **(B)** in R4 and R3, respectively. ^∗^ indicates unknown family of *Acidimicrobiales*, ^∗∗^ indicates *Xanthomonadaceae* and unknown family of *Xanthomonadale*.

The diversity and community structure of methanotrophic enrichment cultures were assessed based on 16S rRNA gene amplicons and are summarized in **Supplementary Table S2** and **Figure 1**. In **Figure 1A**, BLAST-based comparison of the 16S rRNA gene sequence reads with the entries in the SILVA database indicated that members of the family *Methylocystaceae* (15.3%), the family *Xanthomonadaceae* (14.6%), and the family *Acidobacteriaceae* (8.7%) were the most abundant microbial groups in R4. In contrast, the most abundant groups of sequences retrieved from R3 were affiliated with *Acidobacteriaceae* (14.7%), *Chitinophagaceae* (12.2%), an unknown family of *Acidimicrobiales* (10.5%), *Holophagaceae* (10.3%), *Methylocystaceae* (8.1%), and *Porphyromonadaceae* (7.3%). In terms of methanotrophy (shown in **Figure 1B**), members of the families *Methylocystaceae* and *Methylococcaceae* were abundant in both enrichments while members of the family *Beijerinckiaceae* were rare (<0.1%). The higher relative abundance of the family *Methylocystaceae* than that of *Methylococcaceae* in both enrichment cultures (**Figure 1B**) is consistent with the results of previous *in situ* or microcosm studies (Lau et al., 2007; Kip et al., 2011; Sharp et al., 2014; Esson et al., 2016). The families *Methylocystaceae, Methylococcaceae*, and *Beijerinckiaceae* were represented by the members of the genera *Methylocystis, Methylobacter*, and *Methylocapsa*, respectively (**Figure 1B**). Alphaproteobacterial type II methanotrophs, including *Methylocystis* and *Methylocapsa*, have been detected in various acidic ecosystems and several have been isolated (Dedysh et al., 2002, 2007; Belova et al., 2013). Diverse *Methylobacter* species have also been detected in various acidic environments (Kolb et al., 2005; Kip et al., 2011).

Considering the relative abundance of methanotrophs in the original soils based on quantitative PCR of *pmoA* (**Supplementary Table S1**), methanotroph populations were enriched about 10-folds during laboratory incubations. The low relative abundance of methanotrophs (20.5 and 10.5%) in the enrichment cultures, compared with that under neutral pH conditions (Kim et al., 2018), might be caused by slow growth of methanotrophs in acidic conditions (**Supplementary Figure S1**) and using SBR for enrichment method for retaining most of the settle-able biomass. The slow growth of methanotrophs in acidic conditions could be further indicated by the lower abundance of methanotrophs at pH 3 (10.5%) than that pH 4 (20.5%). However, the relative abundance of individual genera (as a proportion of methanotrophs) did not significantly differ between treatments. Non-methanotrophs were more abundant at pH 3 than at pH 4 while the compositions were not significantly different from each other. Several studies have suggested association mechanisms between methanotrophs and non-methanotrophs, such as cross-feeding intermediates of methane metabolisms to non-methanotrophs (Oshkin et al., 2015; Krause et al., 2017) and supplying growth factors (e.g., cobalamin, vitamin B_12_) for methane oxidation from non-methanotrophs (Iguchi et al., 2011).

### Genome Reconstruction

To gain genomic insight into acid adaptation of methanotrophs, genomes were reconstructed from metagenomic sequence reads (R4: 2.3 Gbp Illumina reads and 1.1 Gbp Pacbio reads, R3: 2.7 Gbp Illumina reads and 0.9 Gbp Pacbio reads) and the metabolic and physiological properties of the encoded proteins related to acid adaptation were investigated. Assembled scaffolds from the two enrichment cultures were binned using coverage and tetranucleotide frequency (**Supplementary Figure S2**). Three methanotroph genomes and two genomes affiliated to *Beijerinckiaceae* were reconstructed and the features are summarized in **Table 1**. The completeness of the genomes ranged from partial to near complete (69.83–99.37%) as estimated by using CheckM analysis (Parks et al., 2015). The genome belonging to the family *Methylococcaceae* was designated KS41. Genomes designated KS32 and KS42 were affiliated with the family *Methylocystaceae* and ANI between KS32 and KS42 was >99% suggesting that both genomes represent identical or nearly identical organisms. Genomes of methanotrophs belonging to the family *Beijerinckiaceae* were not obtained. Although phylogenetic analysis of ribosomal protein genes indicated that the genomes KS37 and KS44 were affiliated with the family *Beijerinckiaceae*, no genes encoding either pMMO or sMMO were found. Genome bins affiliated with the following non-methanotrophic clades were also retrieved: *Phenylobacterium* of *Alphaproteobacteria, Rhodanobacter* of *Gammaproteobacteria, Granulicella* of *Acidobacteria, Frankia* of *Actinobacteria*, and *Mucilaginibacter* and *Cytophagales* of *Bacteroidetes*. Therefore, KS32 of *Methylocystaceae* and KS41 of *Methylococcaceae* were the key methanotrophs in the enrichment cultures and further analyses focused exclusively on these two genomes.

**Table 1:** Features of the five reconstructed genome bins obtained through analysis of metagenomic sequences derived from the enrichment cultures R4 and R3.

Genomic bin	KS41	KS32	KS42	KS37	KS44
Bin size (Mb)	4,74	3,36	2,81	2,79	3,09
Number of contigs	4	7	42	27	206
Completeness (%)	99.37	94.36	90.28	92.89	69.83
Contamination	2.23	0.00	0.00	1.41	13.17
Strain heterogeneity	0.00	0.00	0.00	0.00	0.00
Average contig size (kb)	1,185	479	66	103	15
Average GC (%)	46.80	61.27	61.18	62.25	67.59
Number of predicted CDSs	4,226	3,104	2,598	2,752	1,101
Number of genes with assigned function	2,824	2,184	1,716	1,792	564
Number of predicted CDSs (Pfam)	3,314	2,500	2,057	2,284	771
Number of predicted CDS (COG)	2,219	1,848	1,558	1,777	540
Number of genes with assigned hypothetical protein	1,238	813	740	886	409
tRNA	44	47	41	31	16
16S_23S_5S rRNA	3_3_3	2_2_2	2_2_2	0_0_1	ND
Phylogenetic affiliation	*Methylobacter*	*Methylocystis*	*Methylocystis*	*Beijerinckiaceae*	*Beijerinckiaceae*
Metagenome	R4	R3	R4	R3	R3


*Completeness, contamination, and strain heterogeneity were assessed with CheckM.*

### Genomic and Phylogenetic Properties

Publicly available genomes of methanotrophs closely related to KS32 and KS41 were retrieved for comparative genomics (**Supplementary Table S3**). Genome sizes, G+C contents, and number of CDSs of KS32 and KS41 were similar to the reference genomes. The majority of CDSs from KS32 were most similar to homologs in *Methylocystis* spp. (*Methylocystis parvus* OBBP^T^, *Methylocystis* sp. ATCC 49242, and *Methylocystis rosea* SV96^T^: 53% of total CDSs). Likewise, the majority of CDSs from the KS41 genome were most similar to homologs in *Methylobacter* spp. (*M. tundripaludum*: 35.3% of total CDSs; *Methylobacter luteus* IMV-B-3098, *Methylobacter marinus* A45^T^, and *Methylobacter whittenburyi* ACM-3310: 15.56% of total CDSs).

**FIGURE 2:**
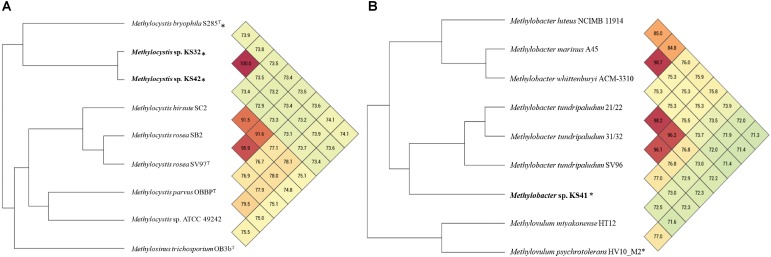
Heatmap and phylogenomic tree based on average nucleotide identity (ANI) values calculated from Orthologous Average Nucleotide Identity Tool (OAT) software of the genome bins of KS42, KS32 **(A)**, KS41 **(B)**, and other reference species. Values larger than 95% indicate that strains belong to the same species. ^∗^ indicates acid-tolerant/acidophilic strains.

Phylogenetic trees based on the 16S rRNA gene and *pmoA* gene (encoding the beta-subunit of pMMO) were constructed in order to infer the phylogenetic relationships of the KS32 and KS42 to their close relatives. Both KS32 and KS41 genome bins contained *pmoCAB* operons while genes encoding sMMO were absent. Additionally, KS41 also contained the genes for *pxmABC*, the homolog of the *pmoCAB*, encoding a copper-containing membrane monooxygenase of unknown function (Tavormina et al., 2011; Hamilton et al., 2015). Based on the 16S rRNA gene and PmoA phylogenetic trees (**Supplementary Figure S3**), KS32 was most closely related to members of *Methylocystis* spp. and formed a monophyletic group with *M. heyeri* H2^T^ and Sakb1 which were isolated from acidic soil environments. KS32 was most similar to *M. heyeri* H2^T^ (99.1% 16S rRNA identity), although genomes for *M. heyeri* are not currently available for further comparison. The 16S rRNA gene similarities between KS32 and other type strains of the genus *Methylocystis* were high. These included strains *M. bryophila* H2s^T^ (97.9%), *M. rosea* SV97^T^ (97.7%), *M. hirsuta* CSC1^T^ (97.6%), *M. parvus* OBBP^T^ (97.2%), and *M. echinoides* IMET 10491 (97.2%). However, the genomes of these type strains share low ANI (72.9–73.9%) with KS32 and KS42 (**Figure 2A**). Thus, KS32 and KS42 are likely new members of *M. heyeri*.

**FIGURE 3:**
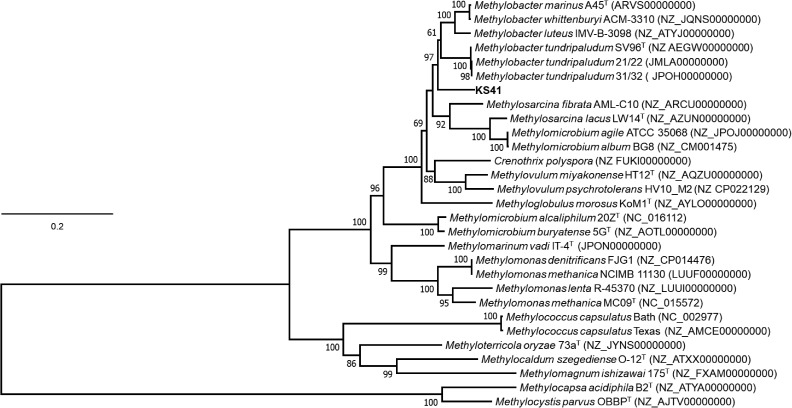
Phylogenetic tree based on a concatenation of 15 syntenic ribosomal proteins of different members of type I methanotroph. The phylogenetic analysis was implemented using the maximum-likelihood method using the Le Gascuel model based on a concatenation of 2467 amino acids total length with 1000 bootstraps. Bootstrap values of >60% from the maximum likelihood, respectively, as indicated at branch points. The scale bar represents 0.1 change per amino-acid position. Genome accession numbers are listed in parentheses. Two type-II methanotrophs, *Methylocapsa acidiphila* B2^T^ and *Methylocystis parvus* OBBP^T^, were used as the outgroup. Evolutionary analyses were conducted in MEGA7.

The 16S rRNA gene sequence from KS41 shared 96.2–98.4% identity with those from members of the genus *Methylobacter*, forming a strongly supported clade with *M. tundripaludum* SV96^T^, *M. psychrophilus* Z-0021^T^, and the taxonomically uncharacterized *Methylobacter* sp. strain T20 (**Supplementary Figure S4A**). Phylogenetic trees based on 15 concatenated conserved ribosomal proteins (**Figure 3**) also matched well with the 16S rRNA tree. The *pmoA* gene sequence of KS41 was as similar to the gene sequences from *Methylovulum* (*M. miyakonense* HT12^T^, 88.6%; *Methylovulum* sp. M200, 87.83%; and *M. psychrotolerans* Sph1^T^, 88%) as it was to *Methylobacter* (*Methylobacter* sp. CMS7, 89.18%; *M. psychrophilus* Z-0021^T^, 87.5%; and *M. tundripaludum* SV96^T^, 86.78%) and the PmoA phylogeny was poorly resolved (**Supplementary Figure S4B**). A lack of resolution and a phylogenetic tree based on the concatenated PmoCAB also places KS41 as a sister taxon to *Methylovulum* (**Supplementary Figure S5**) rather than the *Methylobacter* clade. The placement of KS41 in both the 16S rRNA gene and concatenated ribosomal protein phylogenies is not congruent with the single-gene *pmoA* and the concatenated PmoCAB tree, due to the lack of resolution in the latter two phylogenies. Since metagenomic binning can result in misplaced scaffolds, we explored this as a possible explanation for the incongruency between the 16S rRNA gene and PmoCAB phylogenies. Correct assembly and binning were verified by examining coverage and the alignment of raw reads to scaffolds (**Supplementary Figure S6**). The uniform depth of coverages of the scaffolds of KS41 and KS32, including rRNA operons and *pmo/pxm* operons indicate that these genes were not misassembled or assigned to incorrect bins. Further, successful PCR amplification of *pmoCA* and *pmoCAB* of KS41 from metagenomic DNA indicates physical linkage of the genes by correct assembly.

The values of ANI between KS41 and three *Methylobacter* strains (77.0–73.5%) or two *Methylovulum* strains (71.6–72.5%) indicate the closer genomic similarity between KS41 and *Methylobacter* than *Methylovulum* (**Figure 2B**). The ANI threshold of 95–97% was suggested for species circumscription (Tindall et al., 2010; Lee et al., 2015; Rossello-Mora and Amann, 2015; Thompson et al., 2015) and although KS41 and other *Methylobacter* species share over 97% identity on the level of 16S rRNA gene, these organisms represent unique species according to ANI. Altogether, we propose the name “*Ca*. Methylobacter pinensis KS41” for this species.

### Central Metabolism

All genes required for a methanotrophic lifestyle and for the utilization of various nitrogen sources (**Supplementary Table S4**) were identified in KS41 and KS32 and most of these genes reside in the conserved syntenic regions. A single copy of the *pmoCAB* operon is present in genome KS41 while two copies of the *pmoCAB* operon are present in genome KS32. The genes involved in methanol oxidation (PQQ-dependent dehydrogenase) and formaldehyde oxidation (tetrahydromethanopterin-linked pathway) are present in both assembled genomes. Genomes KS41 and KS32 also encode genes to fix carbon via the ribulose monophosphate pathway and serine pathway, corresponding to type I and type II methanotrophy, respectively. Although the genes encoding for enzymes involved in the serine pathway were present in the KS41 genome, the genes encoding for the malyl-CoA/(*S*)-citramalyl-CoA lyase, hydroxypyruvate reductase, and phosphoenolpyruvate carboxylase could not be found. The incomplete serine pathway was reported in the genomes of *Methylobacter* (**Supplementary Tables S3, S4**) as well as *Methylovulum* (Hamilton et al., 2015). Genes involved in glycolysis, the pentose phosphate pathway, and the TCA cycle were also present in both KS41 and KS32.

Genes in each genome encoded for proteins with the potential for assimilation of nitrate and nitrite, hydroxylamine oxidation, nitrogen fixation, and denitrification. Proteins with the ability to transport nitrate/nitrite across the cytoplasmic membrane (NasA) and reduce it to ammonia (NirBD) may be encoded in both KS41 and KS32. Clusters of genes for urea transport and hydrolysis were identified only in KS41. pMMO can convert ammonia to hydroxylamine which is presumably detoxified by hydroxylamine reductase (*hcp*) in KS32 (Wolfe et al., 2002; Cabello et al., 2004). In the case of KS41, a gene for flavohemoglobin (*hmp*) might be involved in the detoxification of nitric oxide (Bonamore and Boffi, 2008; Vekeman et al., 2016).

### Comparative Analysis of COGs

**FIGURE 4:**
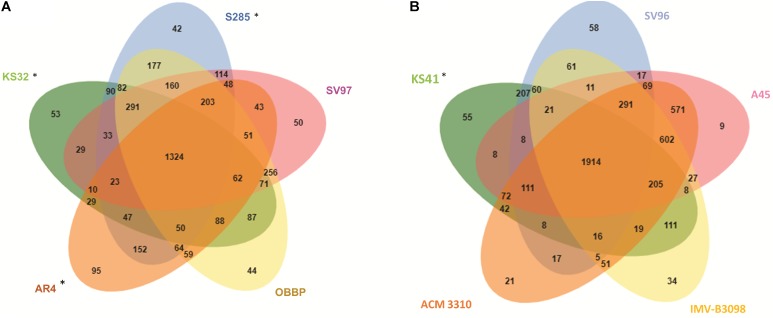
Venn diagrams showing the numbers of shared proteins among *Methylocystis* and *Methyloferula* genus **(A)**, *Methylobacter* genus **(B)**. ^∗^ indicates acid-tolerant/acidophilic strains.

The Venn diagram calculated using OrthoVenn program shows the overlapping orthologous protein clusters between the genomes of KS41 and KS32 and closely related taxa (**Figure 4**). Comparative genome analysis between KS32 and other acidophilic/acid-tolerant strains of *Methylocystis* and *Methyloferula* revealed 3,927 intersecting COGs and 1,324 core COGs (**Figure 4A**). Among the three acidophilic methanotroph genomes, 47 COGs with 169 CDSs (58% hypothetical proteins) were shared (**Supplementary Table S5**). GO-enrichment analysis of the 47 COGs identified several GO-ID including proton-translocating ATP synthase activity (GO:0046933, GO:0046961, GO:0045263, and GO:0042777 group 4 COGs including 12 CDSs) and drug transmembrane transport (GO:0006855 and GO:0015238 group 2 COGs including 7 CDSs) with <0.05 *P*-values, which may indicate mechanisms for growth under acidic conditions.

Comparisons of KS41 with those of neutrophilic *Methylobacter* species including *M. tundripaludum* SV96^T^, *M. whittenburyi* ACM-3310, *M. luteus* IMV-B-3098, and a halophilic strain, *M. marinus* A45^T^ identified 4,709 COGs with functional predictions within the four reference genomes of the genus *Methylobacter* and KS41 as shown in an OrthoVenn-diagram (**Figure 4B**). This analysis predicted 1914 core COGs for all five genomes. KS41 genome shared 2,865 COGs with 3,076 CDSs with those of other *Methylobacter* genomes, sharing the highest number of COGs with a neutrophilic strain, *M. tundripaludum* SV96^T^ (207 COGs with 431 CDSs) (**Supplementary Table S6**). GO-enrichment analysis of KS41-specific genes identified GO-IDs for cellular response to acidic pH (GO:0071468 with 1 COG containing two genes). GO analysis indicated that many genes for genomes KS41 and KS32 were involved in acid adaptation and the genes are shared with other acidophilic/acid-tolerant microorganisms.

### Features of Genomes That Support Acid Adaptation

Complex effects of the proton concentration gradient across the membrane as well as an increased availability of toxic substrates such as heavy metal ions are stressful to microorganisms in acidic environments (Baker-Austin and Dopson, 2007). Comparative genomic analysis of the genomes recovered by us with those of related methanotrophs revealed potential mechanisms adopted by acidophilic methanotrophs for maintaining pH homeostasis and adaptation to various stresses in acidic environments. As summarized in **Supplementary Table S7**, KS32 and KS41 contained 232 genes involved in acid adaptation and shared 91 genes based on KEGG. However, blast analysis indicates that the genes of KS32 and KS41 had the highest similarity to different organisms indicating that no gene was closely related to each other. Most of the genes involved in acid adaptation in KS32 are not specific to acidophilic methanotrophs, while some of the genes involved in acid adaptation in KS41 are related to *M. miyakonense* HT12^T^, as indicated in **Supplementary Table S4**. This might be caused by a scarcity of genomic information on acidophilic methanotrophs. In fact, only one acidophilic *Methylocystaceae* genome (*M. bryophila* S285) genome and two acid-tolerant *Methylococcaceae* genomes (two genomes from *Methylovulum*) genomes are available in a public database.

### KS32

Acid-resistance mechanisms of KS32 include using ion transporter systems for pumping potassium or other cations into cells and pumping protons out of the cell. An extensive array of potassium uptake systems such as a Kup-type low affinity potassium transporter, Trk-, Kch-, and Kef-type potassium transporters, and a Kdp-type high-affinity potassium transporter were found in KS32 (**Supplementary Table S7**). The potassium uptake-system has been shown to also transport ammonium ions which may effectively regulate cytoplasmic pH (Buurman et al., 1991). Genes encoding the high-affinity ATP-driven potassium transporter (*kdpFABC*) are located downstream of two osmo-sensitive signal transduction histidine kinases (*kdpD*) and a response regulator (*OmpR*). Proton exporters also play a primary role in regulating cytoplasmic pH (Baker-Austin and Dopson, 2007) and several homologs of a sodium/proton antiporter gene (*nhaD*) were found in KS32, which may function to export excess protons and simultaneously import sodium ions (Herz et al., 2003). Interestingly, KS32 carries two copies of the F1F0-type ATP synthase genes (**Supplementary Table S4**), which was observed in the genome of an acidophilic verrucomicrobial methanotroph, *M. infernum* (Hou et al., 2008). While one copy is most similar to that in *Methylocystis*, the other is most similar to those from non-methanotrophic *Alphaproteobacteria*. Since ATP synthase is reversible, it is tempting to suggest that the presence of two operons of ATP synthase genes might be associated with pH homeostasis. The genome of KS32 also encoded genes for a metabolic pathway for hopanoid synthesis (**Supplementary Table S7**) which may be important for adapting membranes for life in acidic environments (Jones et al., 2012). Many proteins are involved in the protection and repair of macromolecules damaged by acid stress. Molecular chaperons such as *dnaK* and *groEL* were found in genome KS32, which may prevent periplasmic-protein aggregation under acidic conditions (Merrell and Camilli, 2002; Tucker et al., 2002; Cotter and Hill, 2003).

### KS41

KS41 contains more potassium uptake systems than KS32 (**Supplementary Table S7**) and encodes a gene for a putative chloride channel protein, EriC, of the CLC voltage-gated chloride channel family. EriC protein may remove Cl^-^ (or other suitable anions) from the cell thereby preventing hyperpolarization (Foster, 2004). KS41 contains a gene cluster for urea assimilation including a urease and an ABC transporter for urea. Urea hydrolysis increases pH and thus can be used for acid resistance as suggested in *Helicobacter pylori* (Stingl et al., 2002; Scott et al., 2010). Unexpectedly, the phylogenetic analysis of urea transporter proteins (UrtABCDE proteins, **Supplementary Figure S7A**) and potassium transporter proteins (KdpFABCD, **Supplementary Figure S7B**) indicates that the genes are clustered to a clade containing acidophilic/acid tolerant *Methylovulum* species rather than those of *Methylobacter* species, similar to the results from the phylogenetic analysis of PmoCAB (**Supplementary Figure S5**). In the genomes of neutrophilic *M. tundripaludum*, neither genes for urea uptake nor urea hydrolysis were found. One potential explanation for this finding is that KS41 lost the genes for the urea transporter and acquired the genes from the clade harboring acidophilic *Methylovulum.*

KS41 harbors a cluster of genes encoding for bacterial V-type ATP synthase as well as that for F1F0-type ATP synthase (**Supplementary Table S4**). Such a V-type ATP synthase was not detected in the genome of *M. tundripaludum* strains. The V-type ATP synthase operon is the most similar to that of *Planctomycetes* (such as *Planctomyces brasiliensis, Rhodopirellula maiorica*), and, thus, is likely to have been acquired by a lateral gene transfer event, which could be supported by a phylogenetic tree of the transmembrane C/K subunits of KS41, another methanotroph, and *Planctomycetes* known specifically to be V- or F-type (**Supplementary Figure S8A**). V-type ATP synthases are widely observed in acidophiles which maintain pH homeostasis (Lolkema et al., 2003; Forgac, 2007) by coupling ATP hydrolysis with proton export. A hydrophobic amino acid (A_560_) in the ATP/ADP-binding site may favor proton pumping activity and selectivity of proton/sodium-binding sites of ATP synthase is largely set by the balance of flanking polar and hydrophobic groups (Murata et al., 2008; Schlegel et al., 2012; Leone et al., 2015). For KS41, key hydrophobic residues associated with the ATP/ADP-binding site (**Supplementary Figure S8B**) and the binding site of proton/sodium (**Supplementary Figure S8C**) suggest that the V-type ATPase functions as a proton pump. However, since conserved motifs of V-type ATP synthases are similar in neutrophilic, halophilic, and acidophilic methanotrophs, the roles of these V-type ATP synthase in pH homeostasis need to be verified in the future.

The genome of KS41-encoded genes to synthesize cyclopropane-fatty-acyl-phospholipids (*cfa*) that protect the cell from proton influx under acidic conditions (Mangold et al., 2013b; Lund et al., 2014; Muhling et al., 2016). KS41 also contained genes for starvation-inducible outer membrane lipoprotein (Slp) which is often co-expressed with other acid resistance genes (Foster, 2004; Hou et al., 2008). Interestingly, two putative genes encoding homologs of a molecular chaperon, HdeA, which supports acid resistance in *E. coli* (Gajiwala and Burley, 2000) were also identified in KS41.

The phylogeny of genes providing several key mechanisms for adaptation to acidic environment, particularly those focused on modification of membrane lipids and transport proteins such as KdpFABCD, UrtABCDE, and NtpC of bacterial V-type ATP synthase of KS41 is inconsistent with that of the 16S rRNA.

### Heavy Metal Resistance and Response to Oxidative Stress

Due to the increased solubility of various heavy metals at acidic pH (Johnson, 1998; Nordstrom and Alpers, 1999; Nordstrom et al., 2000), acidophilic methanotrophs must have defense mechanisms against heavy metal toxicity. Both KS32 and KS41 genomes encode elaborate systems for heavy metal efflux pumping systems which are frequently observed in other acidophilic microorganisms: Czc heavy metal efflux pump (resistance–nodulation–cell division transporters, RND) (Baker-Austin et al., 2005), ZntA metal-transporting ATPase, AcrAB multi-drug efflux pump (Mangold et al., 2013a), and a major facilitator superfamily MFS/drug resistance MFS transporter (**Supplementary Table S7**). In addition, genes for copper resistance (*copCD*) and tellurite resistance (*terB*), suggested to be involved in metal homeostasis (Dopson and Holmes, 2014), were identified. Genes for arsenate reductase (*arsC* along with *arsRCDA*) and mercuric reductase (*merA*), which are involved in speciation of metals into less toxic forms (Dopson and Holmes, 2014), were linked to efflux pumps for arsenite (*arsB*) and mercury (*merT* mercuric transporter along with *merRTPA*), respectively. Additionally, Clp genes were found (*clpB* and *clp XP*) which may unfold and degrade aggregated proteins as an adaptation to acidic conditions (Mogk et al., 2003; Sugimoto et al., 2006). An acriflavin resistance protein (AcrB) which is involved in protection from hydrophobic inhibitors (Singh et al., 2010) was found. KS32 contains an additional high-affinity ZnuABC to transport zinc. Cellular damage from high concentrations of toxic substances is frequently associated with the increased production of reactive oxygen species (Silver and Phung, 1996). Both genomes contain an extensive array of response genes for anti-oxidative stresses (**Supplementary Table S7**).

### CRISPR-Cas

**FIGURE 5:**
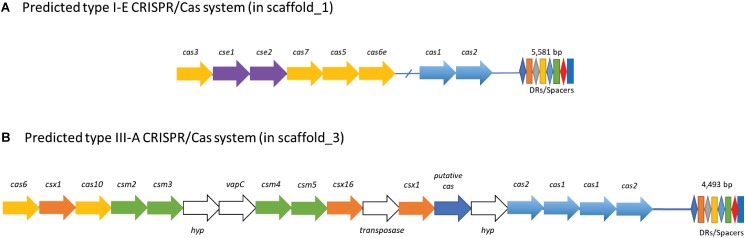
Proposed CRISPR/Cas systems in KS41. KS41 contains **(A)** a putative subtype I-E and **(B)** a putative subtype III-A CRISPR/Cas system. The type I-E CRISPR/Cas system has genes encoding for Cas1/Cas2 and Cas3 proteins, and accessory proteins Cse1/Cse2, Cas7, Cas5, and Cas6e. The type III-A system has a cluster of genes for Cas6, Cas10, and repeat-associated mysterious proteins (RAMPs, Csm2—Cms6), a putative Cas protein. The system has genes coding for one transposase protein and two hypothetical proteins (*hyp*) with unknown functions.

CRISPR-Cas systems are widespread across acidophilic archaea and bacteria and are involved in antiviral defense (Rath et al., 2015; Quatrini et al., 2016). Interestingly, KS41 has 2 CRISPR loci with 154 spacers (**Figure 5**) as well as the corresponding Cas genes (**Supplementary Table S8**). Two different types of CRISPR-Cas systems were also detected in the genome of *M. tundripaludum* SV60^T^ (**Supplementary Table S8**). We could not detect any CRISPR-like sequence in the genome of KS32 or in the phylogenetically related acidophilic methanotrophs, *Methylocystis* and *Methyloferula*. According to the classification and nomenclature of CRISPR-associated genes, KS41 contains type I-E containing *cas123* (Makarova et al., 2011) and type III-A containing *cas12, cas6, cas10*, and *csm2345* (Makarova et al., 2011; Samai et al., 2015). The type I-E CRISPR-Cas system consists of Cas genes which are more closely related to those of acidophilic *Methylovulum miyakonense* strain than those of *Methylobacter* with a high conservation of gene organization and nucleotide identities in *cas3* (94%), *cse1* (53%), *cse2* (62%), and *cas5* (94%). The III-A CRISPR-Cas system contains additional *csx1* and *csx16* of the type III-U system and two copies of *cas1/cas2* which are observed in the genome of an acidophilic iron- and sulfur-oxidizing bacterium *Acidithiobacillus ferrivorans* strain YL15 (Peng et al., 2017). Type I-E and type III-A CRISPR-Cas systems have different viral targets: type I-E targeting viral DNA and type III-B targeting both viral DNA and RNA, respectively (Makarova et al., 2011). Thus, two different CRISPR Cas systems with a large number of spacers may increase resistance to viral infection, which indicates that viral predation may be a key ecological pressure for the survival of *Methylobacter* sp. in acidic conditions.

**FIGURE 6:**
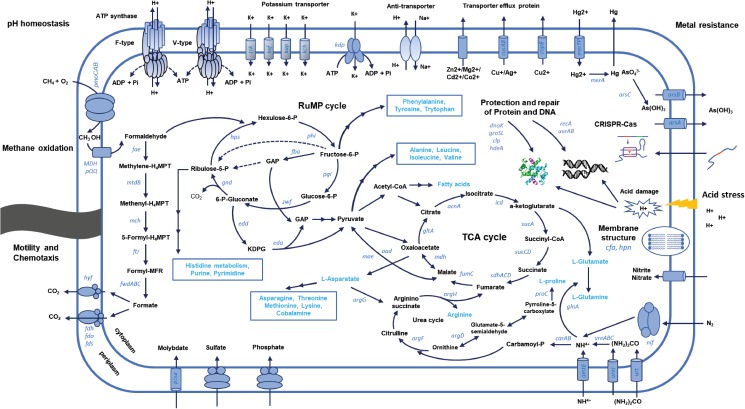
Schematic representation of the metabolic pathways and processes involved in the adaptation of KS41 to acidic condition.

Vast areas of forest soils are experiencing acidification, and acidic wetlands and peat bogs are ecological hot spots because of climate change. Many ecological studies on acidophilic proteobacterial methanotrophs have been conducted in these environments (Lau et al., 2007; Kip et al., 2011; Baesman et al., 2015). So far, studies on adaptation of proteobacterial methanotrophs to acidic environments on genomic levels are rare. Comparative genomic analysis in this study enabled us to understand mechanisms involved in acid adaptation in proteobacterial methanotrophs. The genes involved in acid adaptation mechanisms are closely related to those of neutrophilic methanotrophs or acidophilic non-methanotrophic proteobacteria. Scarcity of genome information of acidophilic methanotrophs limits further investigation of mechanisms specific to acidophilic methanotrophs. KS41 is a rare model of gammaproteobacterial methanotrophs from acidic soils and distinct features for acid adaptation of KS41 could be revealed based on comparative genomics. A schematic model for selected gene products and processes involved in acid adaptation of novel gammaproteobacterial KS41 has been summarized in **Figure 6** with those for K32 in **Supplementary Figure S9**. The genomic reconstruction-based comparative genomics used in this study is still challenging due to the potential problems with (1) contamination during assembly and binning and (2) incoherence between genotype and phenotype. Thus, mechanisms of acid adaptation of methanotrophs suggested in this study warrants further investigation.

## Conclusion

This study identified mechanisms involved in acid adaptation of methanotrophs using comparative analysis of genomes obtained from pine forest soils. The two dominant genomes affiliated to *Methylocystis* (*Alphaproteobacteria*) and *Methylobacter* (*Gammaproteobacteria*) of type II and type I methanotrophs in SBR had several common features for acid adaptation: (1) Key mechanisms for acid resistance identified involved membrane transport proteins associated with cytoplasmic pH homeostasis and resistance to heavy metals. (2) Other mechanisms, including membrane modification and DNA and protein repair may be employed for adaptation to acid stress. In addition, distinct features were also observed in each genome: KS41 and KS32. The phylogenetic analysis indicated that phylogenies of some of the key genes involved in acid adaptation in KS41 were incongruent with that of 16S rRNA gene. With the increasing numbers of acidophilic methanotroph genomes identified, the significance of the features identified in this study could be assessed. This should be followed by confirmation using pure cultures as well as natural acidic soils.

## Data Accessibility

The metagenomic datasets have been deposited in the NCBI SRA database with BioProject number PRJNA470568 (SRR7135743 and SRR7135742). The assembled genomes of *Methylocystis* sp. KS32 and *Methylobacter* sp. KS41 are deposited at DDBJ/ENA/GenBank under the accessions PHSQ00000000 and PHSP00000000.

## Author Contributions

S-KR conceived this work. N-LN and W-JY performed the cultivation and DNA extraction. Genome analysis was carried out by J-HG, S-JK, S-JP, and CH. J-GK and M-YJ analyzed the sample properties. Manuscript was written by N-LN, S-KR, and CH. All authors read and approved the final manuscript.

## Conflict of Interest Statement

The authors declare that the research was conducted in the absence of any commercial or financial relationships that could be construed as a potential conflict of interest.

## Footnotes

1 https://www.arb-silva.de/ngs
