# Supplementary material for: Genomic Insights Into the Acid Adaptation of Novel Methanotrophs Enriched From Acidic Forest Soils
Source: Front Microbiol. 2018 Aug 27;9:1982. doi: 10.3389/fmicb.2018.01982 (PMC6119699; doi:10.3389/fmicb.2018.01982)
Supplement: Supplementary file 1 [file Presentation_1.PPTX]

## Slide 1
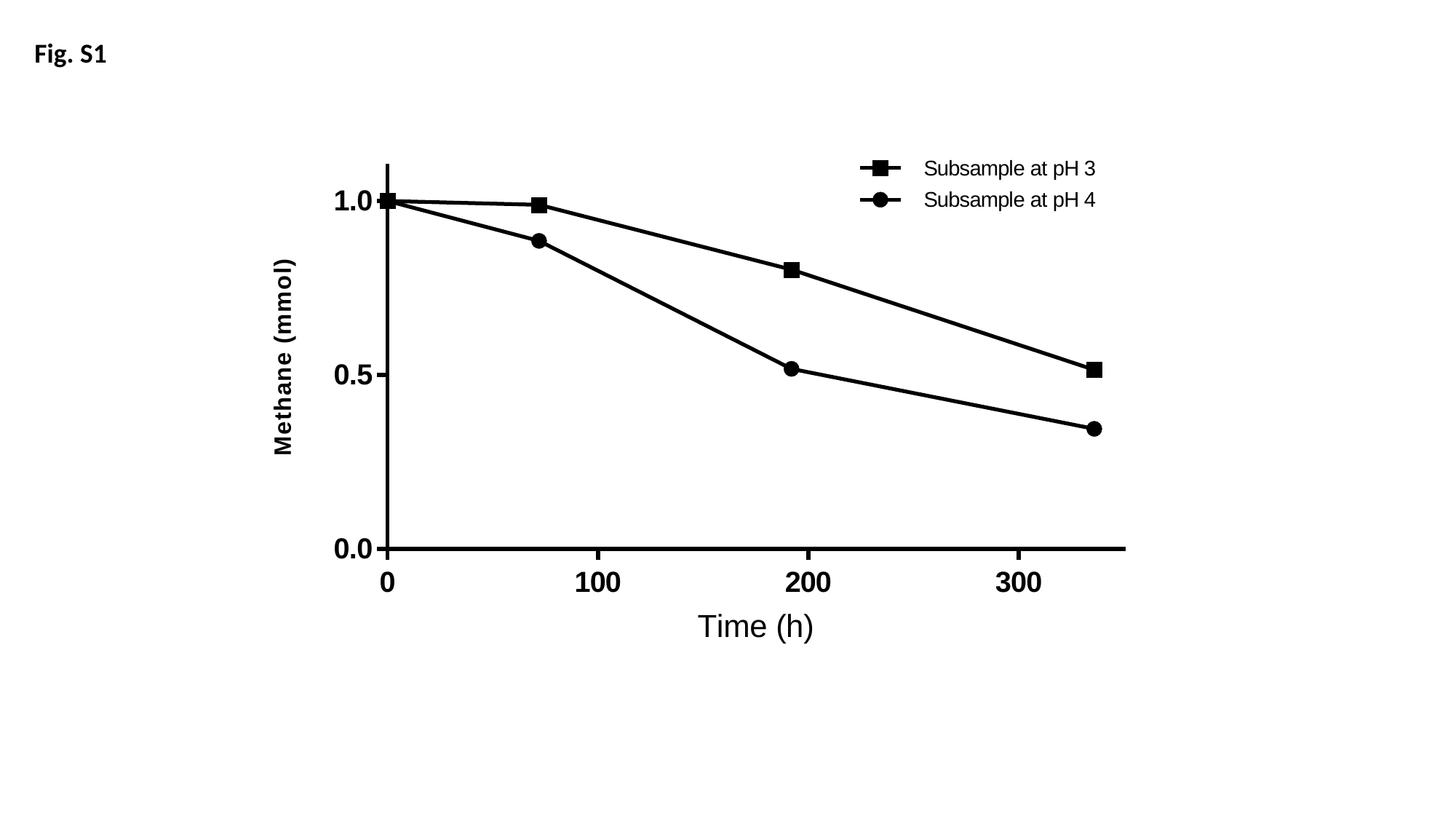

Fig. S1

## Slide 2
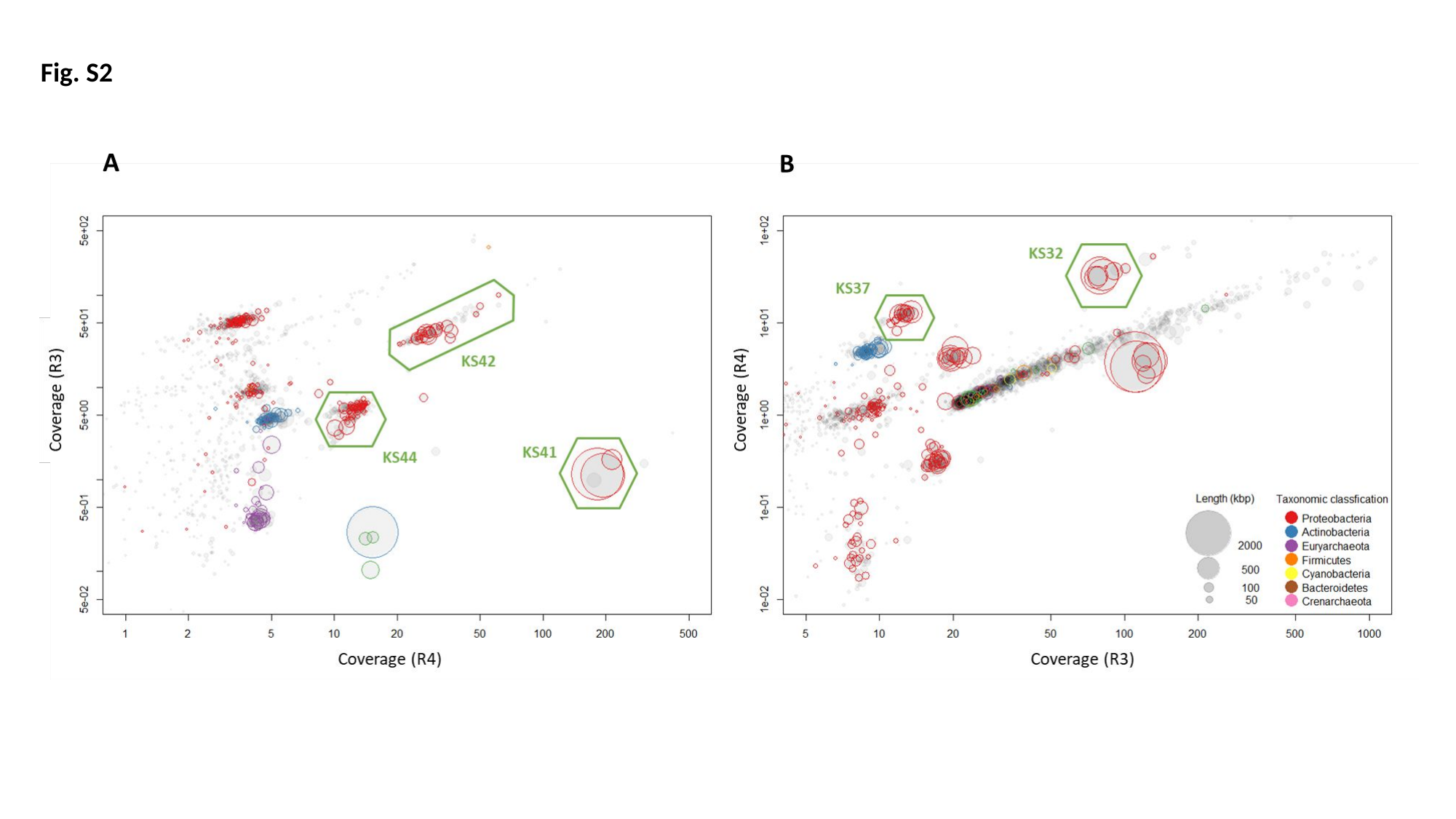

Fig. S2

## Slide 3
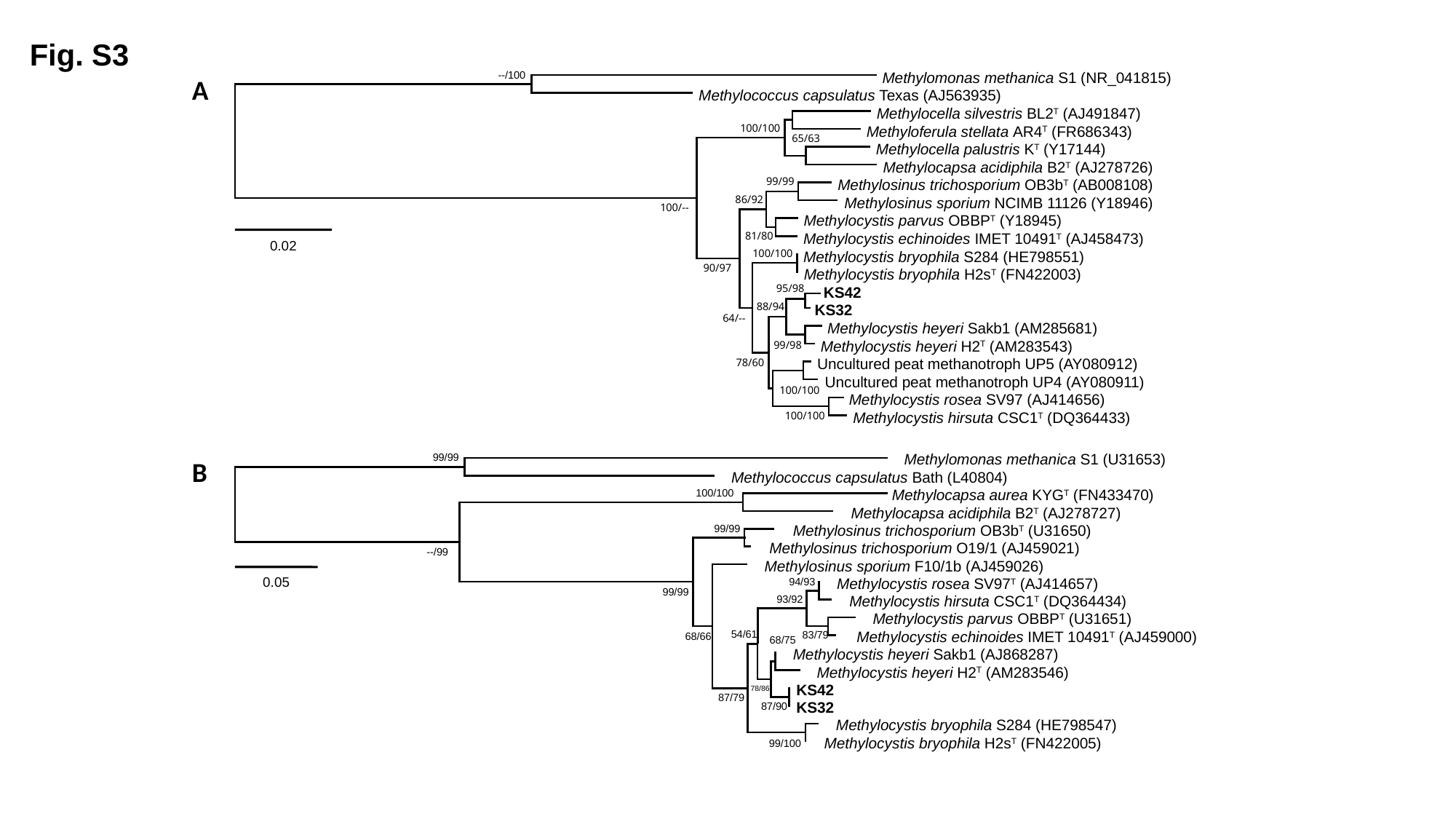

Fig. S3
A
 Methylomonas methanica S1 (NR_041815)
 Methylococcus capsulatus Texas (AJ563935)
 Methylocella silvestris BL2T (AJ491847)
 Methyloferula stellata AR4T (FR686343)
100/100
65/63
 Methylocella palustris KT (Y17144)
 Methylocapsa acidiphila B2T (AJ278726)
99/99
 Methylosinus trichosporium OB3bT (AB008108)
86/92
 Methylosinus sporium NCIMB 11126 (Y18946)
100/--
 Methylocystis parvus OBBPT (Y18945)
 Methylocystis echinoides IMET 10491T (AJ458473)
81/80
0.02
100/100
 Methylocystis bryophila S284 (HE798551)
90/97
 Methylocystis bryophila H2sT (FN422003)
95/98
 KS42
88/94
 KS32
64/--
 Methylocystis heyeri Sakb1 (AM285681)
 Methylocystis heyeri H2T (AM283543)
99/98
 Uncultured peat methanotroph UP5 (AY080912)
78/60
 Uncultured peat methanotroph UP4 (AY080911)
100/100
 Methylocystis rosea SV97 (AJ414656)
 Methylocystis hirsuta CSC1T (DQ364433)
100/100
--/100
B
 Methylomonas methanica S1 (U31653)
99/99
 Methylococcus capsulatus Bath (L40804)
 Methylocapsa aurea KYGT (FN433470)
100/100
 Methylocapsa acidiphila B2T (AJ278727)
 Methylosinus trichosporium OB3bT (U31650)
99/99
 Methylosinus trichosporium O19/1 (AJ459021)
 Methylosinus sporium F10/1b (AJ459026)
0.05
 Methylocystis rosea SV97T (AJ414657)
94/93
99/99
 Methylocystis hirsuta CSC1T (DQ364434)
93/92
 Methylocystis parvus OBBPT (U31651)
 Methylocystis echinoides IMET 10491T (AJ459000)
54/61
83/79
68/66
68/75
 Methylocystis heyeri Sakb1 (AJ868287)
 Methylocystis heyeri H2T (AM283546)
 KS42
78/86
87/79
 KS32
87/90
 Methylocystis bryophila S284 (HE798547)
 Methylocystis bryophila H2sT (FN422005)
99/100
--/99

## Slide 4
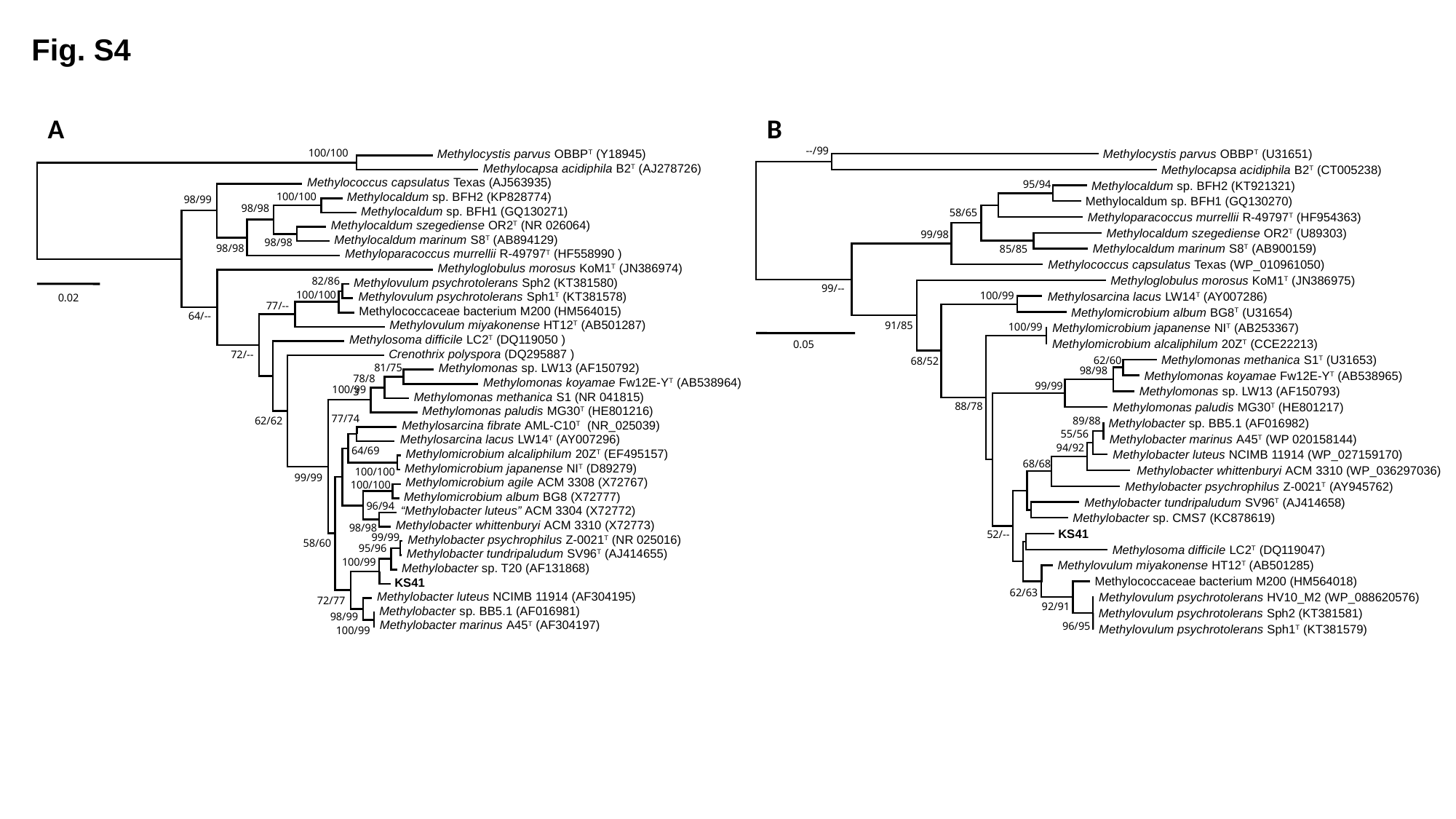

Fig. S4
A
B
--/99
100/100
 Methylocystis parvus OBBPT (Y18945)
 Methylocapsa acidiphila B2T (AJ278726)
 Methylococcus capsulatus Texas (AJ563935)
 Methylocaldum sp. BFH2 (KP828774)
100/100
98/99
98/98
 Methylocaldum sp. BFH1 (GQ130271)
 Methylocaldum szegediense OR2T (NR 026064)
 Methylocaldum marinum S8T (AB894129)
98/98
98/98
 Methyloparacoccus murrellii R-49797T (HF558990 )
 Methyloglobulus morosus KoM1T (JN386974)
82/86
 Methylovulum psychrotolerans Sph2 (KT381580)
100/100
 Methylovulum psychrotolerans Sph1T (KT381578)
0.02
77/--
 Methylococcaceae bacterium M200 (HM564015)
64/--
 Methylovulum miyakonense HT12T (AB501287)
 Methylosoma difficile LC2T (DQ119050 )
 Crenothrix polyspora (DQ295887 )
72/--
 Methylomonas sp. LW13 (AF150792)
81/75
78/83
 Methylomonas koyamae Fw12E-YT (AB538964)
100/99
 Methylomonas methanica S1 (NR 041815)
 Methylomonas paludis MG30T (HE801216)
77/74
62/62
 Methylosarcina fibrate AML-C10T (NR_025039)
 Methylosarcina lacus LW14T (AY007296)
64/69
 Methylomicrobium alcaliphilum 20ZT (EF495157)
 Methylomicrobium japanense NIT (D89279)
100/100
99/99
 Methylomicrobium agile ACM 3308 (X72767)
100/100
 Methylomicrobium album BG8 (X72777)
96/94
 “Methylobacter luteus” ACM 3304 (X72772)
 Methylobacter whittenburyi ACM 3310 (X72773)
98/98
99/99
 Methylobacter psychrophilus Z-0021T (NR 025016)
58/60
95/96
 Methylobacter tundripaludum SV96T (AJ414655)
100/99
 Methylobacter sp. T20 (AF131868)
 KS41
 Methylobacter luteus NCIMB 11914 (AF304195)
72/77
 Methylobacter sp. BB5.1 (AF016981)
98/99
 Methylobacter marinus A45T (AF304197)
100/99
 Methylocystis parvus OBBPT (U31651)
 Methylocapsa acidiphila B2T (CT005238)
95/94
 Methylocaldum sp. BFH2 (KT921321)
 Methylocaldum sp. BFH1 (GQ130270)
58/65
 Methyloparacoccus murrellii R-49797T (HF954363)
 Methylocaldum szegediense OR2T (U89303)
99/98
 Methylocaldum marinum S8T (AB900159)
85/85
 Methylococcus capsulatus Texas (WP_010961050)
 Methyloglobulus morosus KoM1T (JN386975)
99/--
100/99
 Methylosarcina lacus LW14T (AY007286)
 Methylomicrobium album BG8T (U31654)
91/85
100/99
 Methylomicrobium japanense NIT (AB253367)
 Methylomicrobium alcaliphilum 20ZT (CCE22213)
0.05
 Methylomonas methanica S1T (U31653)
62/60
68/52
98/98
 Methylomonas koyamae Fw12E-YT (AB538965)
99/99
 Methylomonas sp. LW13 (AF150793)
88/78
 Methylomonas paludis MG30T (HE801217)
89/88
 Methylobacter sp. BB5.1 (AF016982)
55/56
 Methylobacter marinus A45T (WP 020158144)
94/92
 Methylobacter luteus NCIMB 11914 (WP_027159170)
68/68
 Methylobacter whittenburyi ACM 3310 (WP_036297036)
 Methylobacter psychrophilus Z-0021T (AY945762)
 Methylobacter tundripaludum SV96T (AJ414658)
 Methylobacter sp. CMS7 (KC878619)
 KS41
52/--
 Methylosoma difficile LC2T (DQ119047)
 Methylovulum miyakonense HT12T (AB501285)
 Methylococcaceae bacterium M200 (HM564018)
62/63
 Methylovulum psychrotolerans HV10_M2 (WP_088620576)
92/91
 Methylovulum psychrotolerans Sph2 (KT381581)
96/95
 Methylovulum psychrotolerans Sph1T (KT381579)

## Slide 5
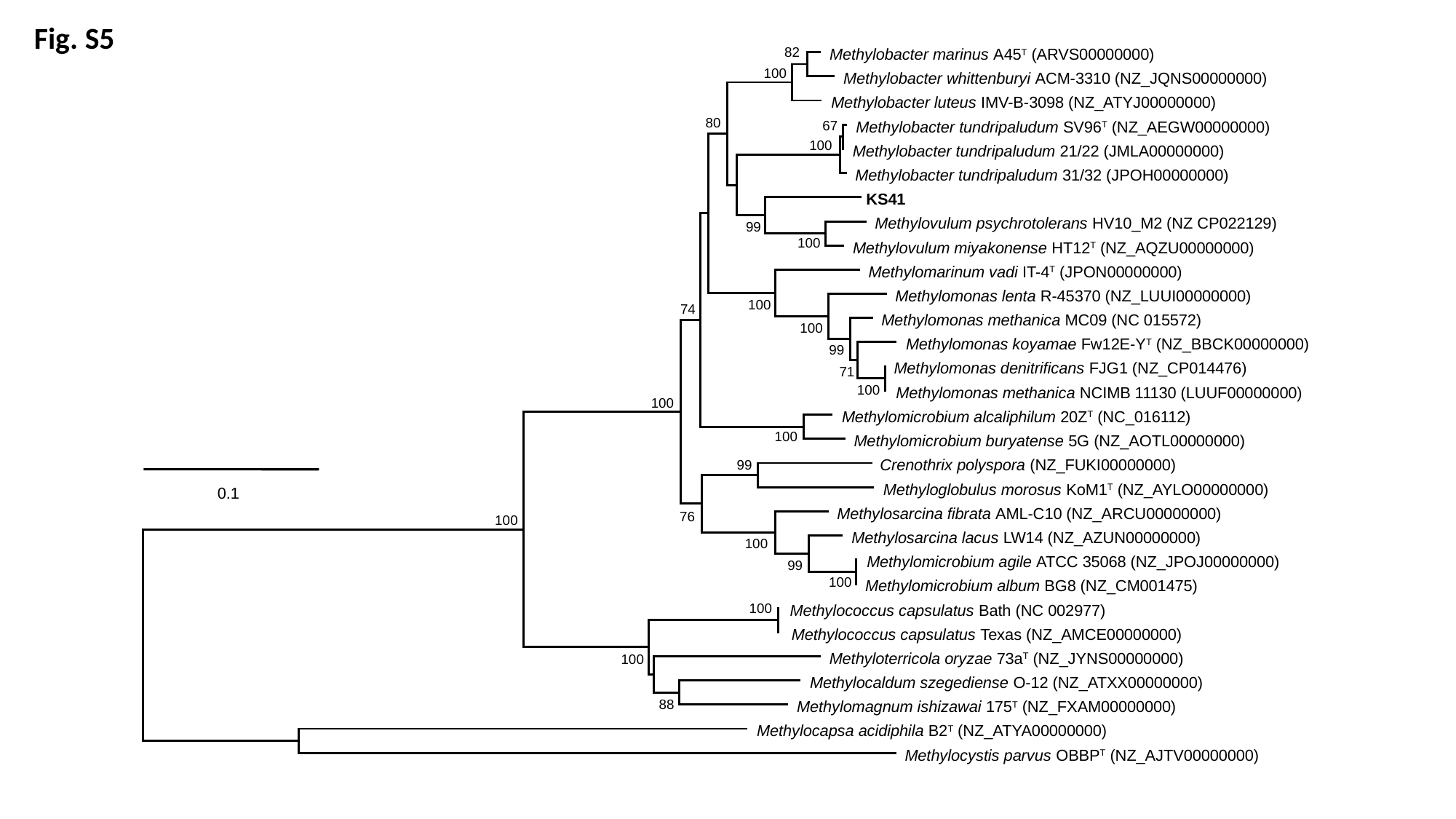

Fig. S5
82
 Methylobacter marinus A45T (ARVS00000000)
100
 Methylobacter whittenburyi ACM-3310 (NZ_JQNS00000000)
 Methylobacter luteus IMV-B-3098 (NZ_ATYJ00000000)
80
 Methylobacter tundripaludum SV96T (NZ_AEGW00000000)
67
100
 Methylobacter tundripaludum 21/22 (JMLA00000000)
 Methylobacter tundripaludum 31/32 (JPOH00000000)
 KS41
 Methylovulum psychrotolerans HV10_M2 (NZ CP022129)
99
100
 Methylovulum miyakonense HT12T (NZ_AQZU00000000)
 Methylomarinum vadi IT-4T (JPON00000000)
 Methylomonas lenta R-45370 (NZ_LUUI00000000)
100
74
 Methylomonas methanica MC09 (NC 015572)
100
 Methylomonas koyamae Fw12E-YT (NZ_BBCK00000000)
99
 Methylomonas denitrificans FJG1 (NZ_CP014476)
71
100
 Methylomonas methanica NCIMB 11130 (LUUF00000000)
100
 Methylomicrobium alcaliphilum 20ZT (NC_016112)
100
 Methylomicrobium buryatense 5G (NZ_AOTL00000000)
 Crenothrix polyspora (NZ_FUKI00000000)
99
 Methyloglobulus morosus KoM1T (NZ_AYLO00000000)
0.1
 Methylosarcina fibrata AML-C10 (NZ_ARCU00000000)
76
100
 Methylosarcina lacus LW14 (NZ_AZUN00000000)
100
 Methylomicrobium agile ATCC 35068 (NZ_JPOJ00000000)
99
100
 Methylomicrobium album BG8 (NZ_CM001475)
100
 Methylococcus capsulatus Bath (NC 002977)
 Methylococcus capsulatus Texas (NZ_AMCE00000000)
 Methyloterricola oryzae 73aT (NZ_JYNS00000000)
100
 Methylocaldum szegediense O-12 (NZ_ATXX00000000)
88
 Methylomagnum ishizawai 175T (NZ_FXAM00000000)
 Methylocapsa acidiphila B2T (NZ_ATYA00000000)
 Methylocystis parvus OBBPT (NZ_AJTV00000000)

## Slide 6
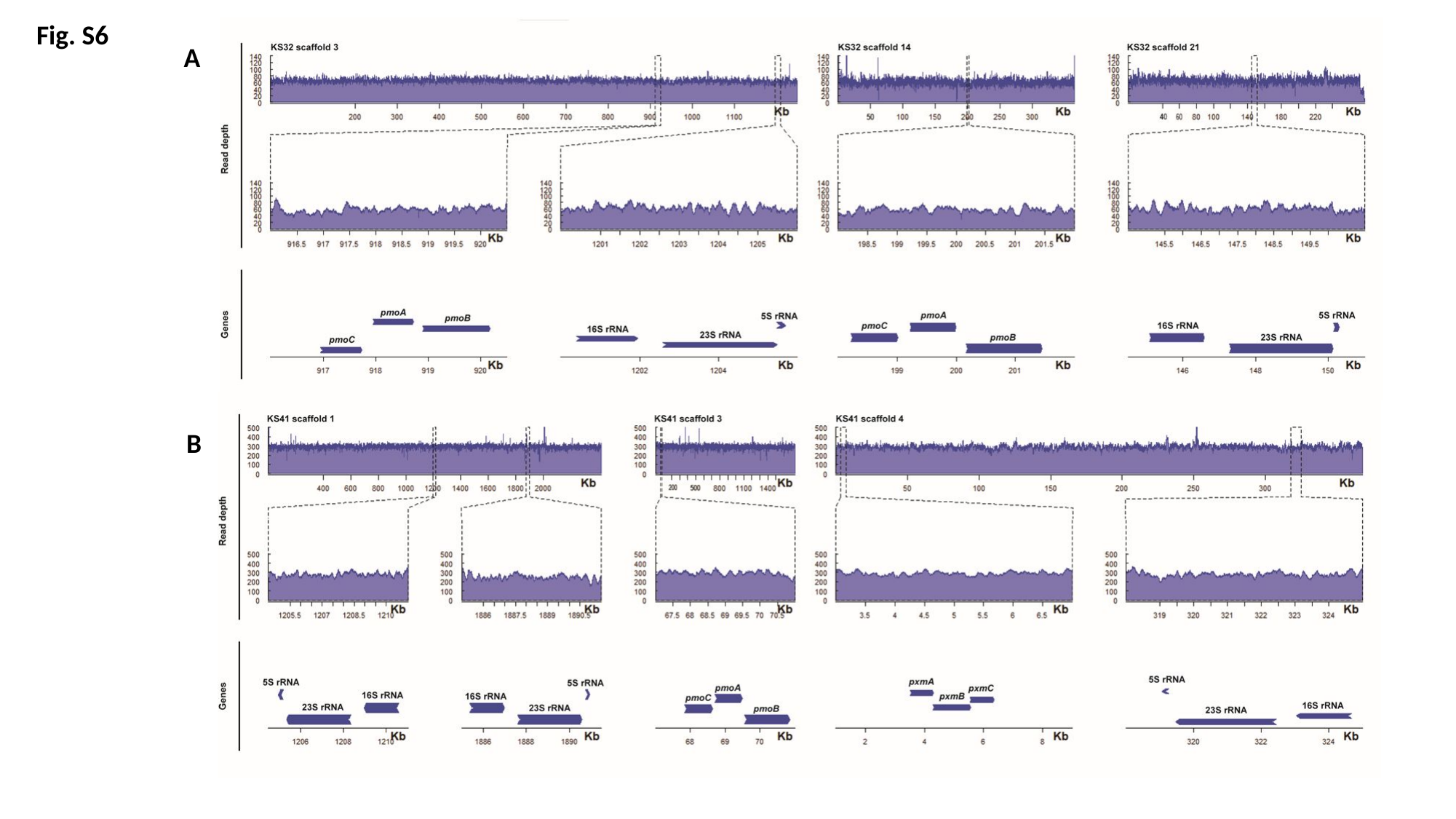

Fig. S6
A
B

## Slide 7
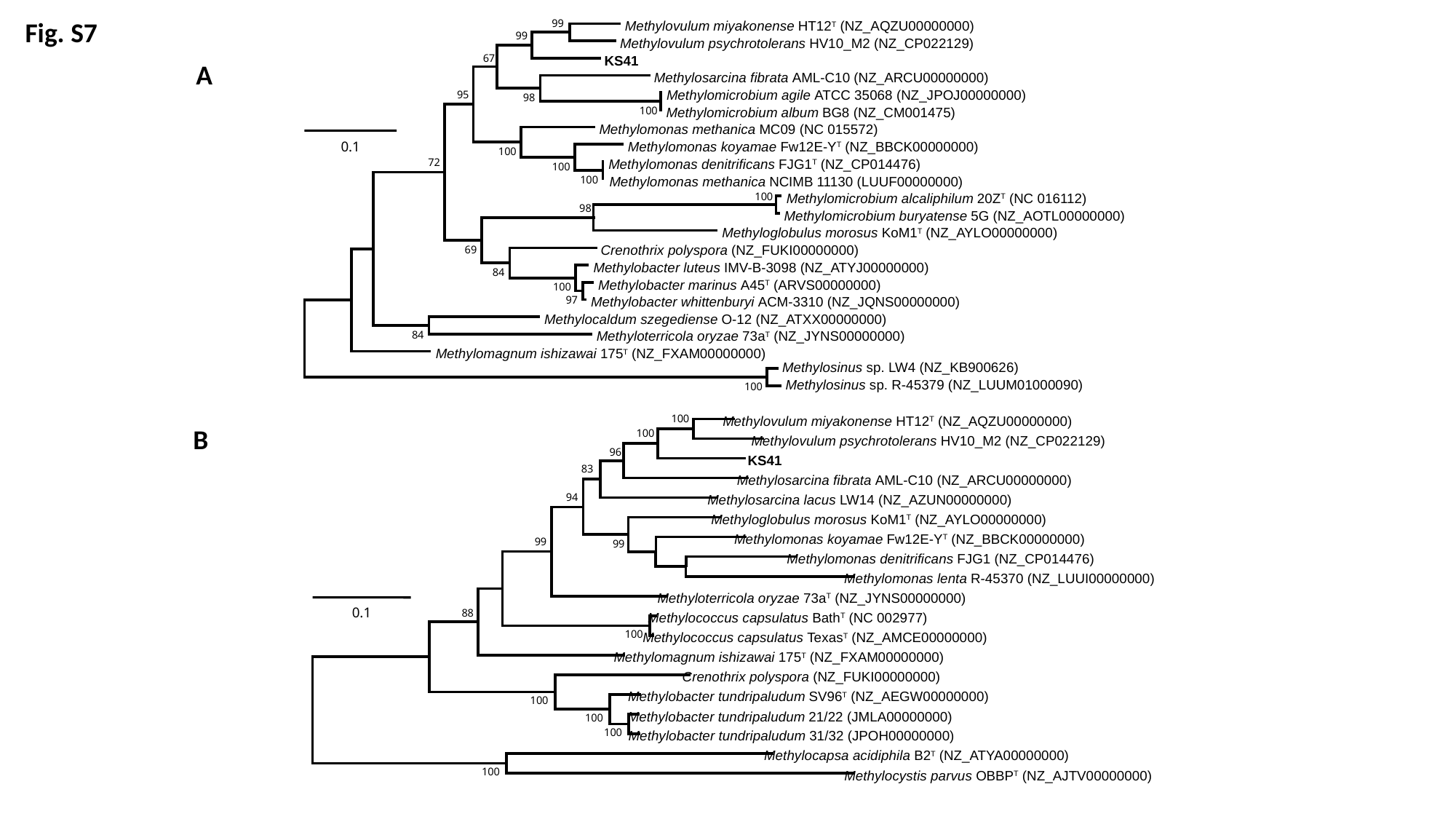

Fig. S7
99
 Methylovulum miyakonense HT12T (NZ_AQZU00000000)
99
 Methylovulum psychrotolerans HV10_M2 (NZ_CP022129)
67
 KS41
 Methylosarcina fibrata AML-C10 (NZ_ARCU00000000)
 Methylomicrobium agile ATCC 35068 (NZ_JPOJ00000000)
95
98
 Methylomicrobium album BG8 (NZ_CM001475)
100
 Methylomonas methanica MC09 (NC 015572)
0.1
 Methylomonas koyamae Fw12E-YT (NZ_BBCK00000000)
100
 Methylomonas denitrificans FJG1T (NZ_CP014476)
72
100
 Methylomonas methanica NCIMB 11130 (LUUF00000000)
100
 Methylomicrobium alcaliphilum 20ZT (NC 016112)
100
98
 Methylomicrobium buryatense 5G (NZ_AOTL00000000)
 Methyloglobulus morosus KoM1T (NZ_AYLO00000000)
 Crenothrix polyspora (NZ_FUKI00000000)
69
 Methylobacter luteus IMV-B-3098 (NZ_ATYJ00000000)
84
 Methylobacter marinus A45T (ARVS00000000)
100
 Methylobacter whittenburyi ACM-3310 (NZ_JQNS00000000)
97
 Methylocaldum szegediense O-12 (NZ_ATXX00000000)
 Methyloterricola oryzae 73aT (NZ_JYNS00000000)
84
 Methylomagnum ishizawai 175T (NZ_FXAM00000000)
 Methylosinus sp. LW4 (NZ_KB900626)
 Methylosinus sp. R-45379 (NZ_LUUM01000090)
100
A
100
 Methylovulum miyakonense HT12T (NZ_AQZU00000000)
100
 Methylovulum psychrotolerans HV10_M2 (NZ_CP022129)
96
 KS41
83
 Methylosarcina fibrata AML-C10 (NZ_ARCU00000000)
94
 Methylosarcina lacus LW14 (NZ_AZUN00000000)
 Methyloglobulus morosus KoM1T (NZ_AYLO00000000)
 Methylomonas koyamae Fw12E-YT (NZ_BBCK00000000)
99
99
 Methylomonas denitrificans FJG1 (NZ_CP014476)
 Methylomonas lenta R-45370 (NZ_LUUI00000000)
 Methyloterricola oryzae 73aT (NZ_JYNS00000000)
0.1
88
 Methylococcus capsulatus BathT (NC 002977)
100
 Methylococcus capsulatus TexasT (NZ_AMCE00000000)
 Methylomagnum ishizawai 175T (NZ_FXAM00000000)
 Crenothrix polyspora (NZ_FUKI00000000)
 Methylobacter tundripaludum SV96T (NZ_AEGW00000000)
100
 Methylobacter tundripaludum 21/22 (JMLA00000000)
100
100
 Methylobacter tundripaludum 31/32 (JPOH00000000)
 Methylocapsa acidiphila B2T (NZ_ATYA00000000)
100
 Methylocystis parvus OBBPT (NZ_AJTV00000000)
B

## Slide 8
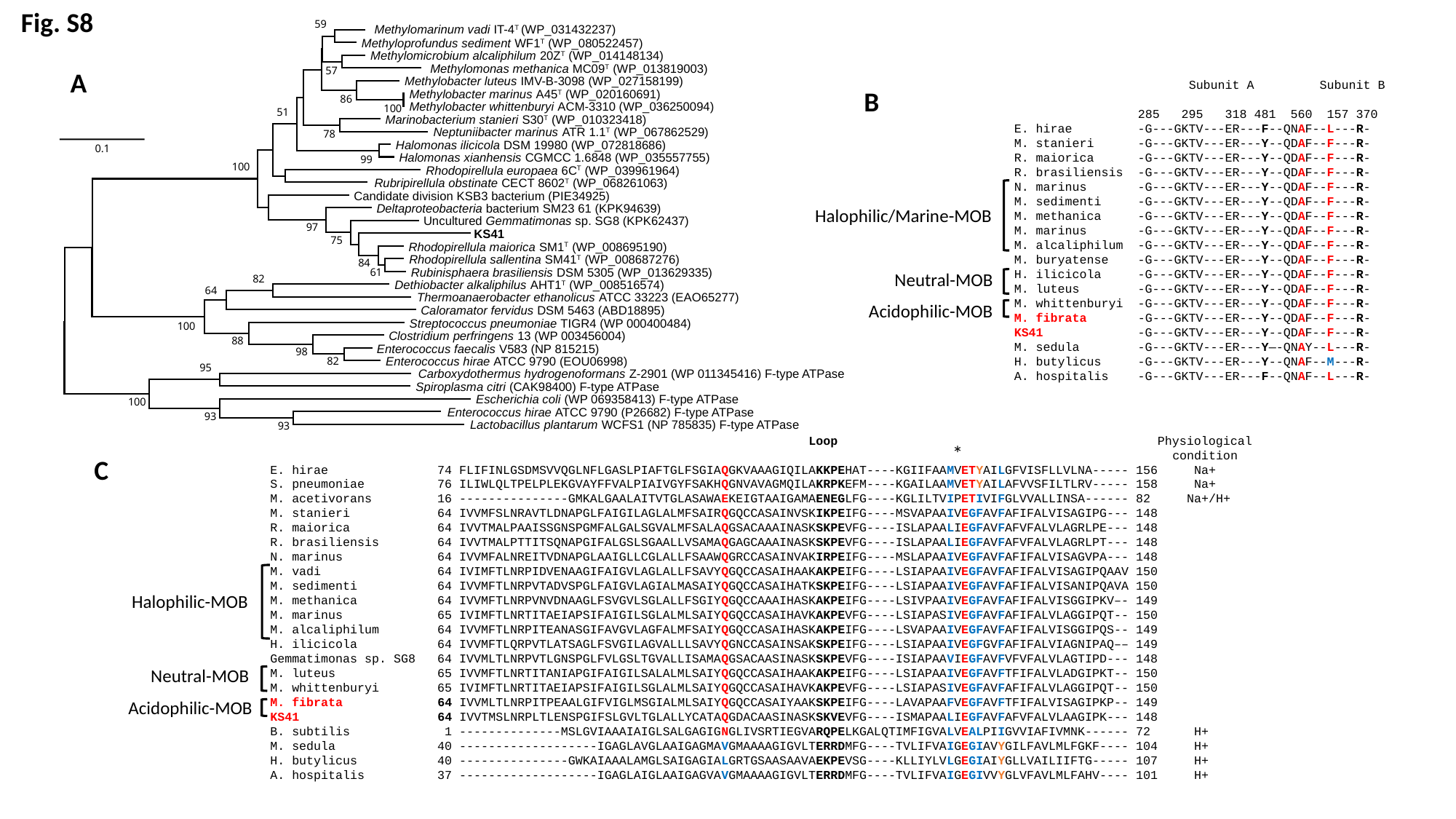

Fig. S8
59
 Methylomarinum vadi IT-4T (WP_031432237)
 Methyloprofundus sediment WF1T (WP_080522457)
 Methylomicrobium alcaliphilum 20ZT (WP_014148134)
 Methylomonas methanica MC09T (WP_013819003)
57
 Methylobacter luteus IMV-B-3098 (WP_027158199)
 Methylobacter marinus A45T (WP_020160691)
86
 Methylobacter whittenburyi ACM-3310 (WP_036250094)
100
51
 Marinobacterium stanieri S30T (WP_010323418)
 Neptuniibacter marinus ATR 1.1T (WP_067862529)
78
 Halomonas ilicicola DSM 19980 (WP_072818686)
0.1
 Halomonas xianhensis CGMCC 1.6848 (WP_035557755)
99
100
 Rhodopirellula europaea 6CT (WP_039961964)
 Rubripirellula obstinate CECT 8602T (WP_068261063)
 Candidate division KSB3 bacterium (PIE34925)
 Deltaproteobacteria bacterium SM23 61 (KPK94639)
 Uncultured Gemmatimonas sp. SG8 (KPK62437)
97
 KS41
75
 Rhodopirellula maiorica SM1T (WP_008695190)
 Rhodopirellula sallentina SM41T (WP_008687276)
84
 Rubinisphaera brasiliensis DSM 5305 (WP_013629335)
61
82
 Dethiobacter alkaliphilus AHT1T (WP_008516574)
64
 Thermoanaerobacter ethanolicus ATCC 33223 (EAO65277)
 Caloramator fervidus DSM 5463 (ABD18895)
 Streptococcus pneumoniae TIGR4 (WP 000400484)
100
 Clostridium perfringens 13 (WP 003456004)
88
 Enterococcus faecalis V583 (NP 815215)
98
 Enterococcus hirae ATCC 9790 (EOU06998)
82
95
 Carboxydothermus hydrogenoformans Z-2901 (WP 011345416) F-type ATPase
 Spiroplasma citri (CAK98400) F-type ATPase
 Escherichia coli (WP 069358413) F-type ATPase
100
 Enterococcus hirae ATCC 9790 (P26682) F-type ATPase
93
 Lactobacillus plantarum WCFS1 (NP 785835) F-type ATPase
93
A
 Subunit A Subunit B
 285 295 318 481 560 157 370
E. hirae -G---GKTV---ER---F--QNAF--L---R-
M. stanieri -G---GKTV---ER---Y--QDAF--F---R-
R. maiorica -G---GKTV---ER---Y--QDAF--F---R-
R. brasiliensis -G---GKTV---ER---Y--QDAF--F---R-
N. marinus -G---GKTV---ER---Y--QDAF--F---R-
M. sedimenti -G---GKTV---ER---Y--QDAF--F---R-
M. methanica -G---GKTV---ER---Y--QDAF--F---R-
M. marinus -G---GKTV---ER---Y--QDAF--F---R-
M. alcaliphilum -G---GKTV---ER---Y--QDAF--F---R-
M. buryatense -G---GKTV---ER---Y--QDAF--F---R-
H. ilicicola -G---GKTV---ER---Y--QDAF--F---R-
M. luteus -G---GKTV---ER---Y--QDAF--F---R-
M. whittenburyi -G---GKTV---ER---Y--QDAF--F---R-
M. fibrata -G---GKTV---ER---Y--QDAF--F---R-
KS41 -G---GKTV---ER---Y--QDAF--F---R-
M. sedula -G---GKTV---ER---Y—-QNAY--L---R-
H. butylicus -G---GKTV---ER---Y--QNAF--M---R-
A. hospitalis -G---GKTV---ER---F--QNAF--L---R-
B
Halophilic/Marine-MOB
Neutral-MOB
Acidophilic-MOB
 Loop Physiological
 condition
E. hirae 74 FLIFINLGSDMSVVQGLNFLGASLPIAFTGLFSGIAQGKVAAAGIQILAKKPEHAT----KGIIFAAMVETYAILGFVISFLLVLNA----- 156 Na+
S. pneumoniae 76 ILIWLQLTPELPLEKGVAYFFVALPIAIVGYFSAKHQGNVAVAGMQILAKRPKEFM----KGAILAAMVETYAILAFVVSFILTLRV----- 158 Na+
M. acetivorans 16 ---------------GMKALGAALAITVTGLASAWAEKEIGTAAIGAMAENEGLFG----KGLILTVIPETIVIFGLVVALLINSA------ 82 Na+/H+
M. stanieri 64 IVVMFSLNRAVTLDNAPGLFAIGILAGLALMFSAIRQGQCCASAINVSKIKPEIFG----MSVAPAAIVEGFAVFAFIFALVISAGIPG--- 148
R. maiorica 64 IVVTMALPAAISSGNSPGMFALGALSGVALMFSALAQGSACAAAINASKSKPEVFG----ISLAPAALIEGFAVFAFVFALVLAGRLPE--- 148
R. brasiliensis 64 IVVTMALPTTITSQNAPGIFALGSLSGAALLVSAMAQGAGCAAAINASKSKPEVFG----ISLAPAALIEGFAVFAFVFALVLAGRLPT--- 148
N. marinus 64 IVVMFALNREITVDNAPGLAAIGLLCGLALLFSAAWQGRCCASAINVAKIRPEIFG----MSLAPAAIVEGFAVFAFIFALVISAGVPA--- 148
M. vadi 64 IVIMFTLNRPIDVENAAGIFAIGVLAGLALLFSAVYQGQCCASAIHAAKAKPEIFG----LSIAPAAIVEGFAVFAFIFALVISAGIPQAAV 150
M. sedimenti 64 IVVMFTLNRPVTADVSPGLFAIGVLAGIALMASAIYQGQCCASAIHATKSKPEIFG----LSIAPAAIVEGFAVFAFIFALVISANIPQAVA 150
M. methanica 64 IVVMFTLNRPVNVDNAAGLFSVGVLSGLALLFSGIYQGQCCAAAIHASKAKPEIFG----LSIVPAAIVEGFAVFAFIFALVISGGIPKV–- 149
M. marinus 65 IVIMFTLNRTITAEIAPSIFAIGILSGLALMLSAIYQGQCCASAIHAVKAKPEVFG----LSIAPASIVEGFAVFAFIFALVLAGGIPQT-- 150
M. alcaliphilum 64 IVVMFTLNRPITEANASGIFAVGVLAGFALMFSAIYQGQCCASAIHASKAKPEIFG----LSVAPAAIVEGFAVFAFIFALVISGGIPQS-- 149
H. ilicicola 64 IVVMFTLQRPVTLATSAGLFSVGILAGVALLLSAVYQGNCCASAINSAKSKPEIFG----LSIAPAAIVEGFGVFAFIFALVIAGNIPAQ–– 149
Gemmatimonas sp. SG8 64 IVVMLTLNRPVTLGNSPGLFVLGSLTGVALLISAMAQGSACAASINASKSKPEVFG----ISIAPAAVIEGFAVFVFVFALVLAGTIPD--- 148
M. luteus 65 IVVMFTLNRTITANIAPGIFAIGILSALALMLSAIYQGQCCASAIHAAKAKPEIFG----LSIAPAAIVEGFAVFTFIFALVLADGIPKT-- 150
M. whittenburyi 65 IVIMFTLNRTITAEIAPSIFAIGILSGLALMLSAIYQGQCCASAIHAVKAKPEVFG----LSIAPASIVEGFAVFAFIFALVLAGGIPQT-- 150
M. fibrata 64 IVVMLTLNRPITPEAALGIFVIGLMSGIALMLSAIYQGQCCASAIYAAKSKPEIFG----LAVAPAAFVEGFAVFTFIFALVISAGIPKP-- 149
KS41 64 IVVTMSLNRPLTLENSPGIFSLGVLTGLALLYCATAQGDACAASINASKSKVEVFG----ISMAPAALIEGFAVFAFVFALVLAAGIPK--- 148
B. subtilis 1 --------------MSLGVIAAAIAIGLSALGAGIGNGLIVSRTIEGVARQPELKGALQTIMFIGVALVEALPIIGVVIAFIVMNK------ 72 H+
M. sedula 40 -------------------IGAGLAVGLAAIGAGMAVGMAAAAGIGVLTERRDMFG----TVLIFVAIGEGIAVYGILFAVLMLFGKF---- 104 H+
H. butylicus 40 ---------------GWKAIAAALAMGLSAIGAGIALGRTGSAASAAVAEKPEVSG----KLLIYLVLGEGIAIYGLLVAILIIFTG----- 107 H+
A. hospitalis 37 -------------------IGAGLAIGLAAIGAGVAVGMAAAAGIGVLTERRDMFG----TVLIFVAIGEGIVVYGLVFAVLMLFAHV---- 101 H+
 *
C
Halophilic-MOB
Neutral-MOB
Acidophilic-MOB

## Slide 9
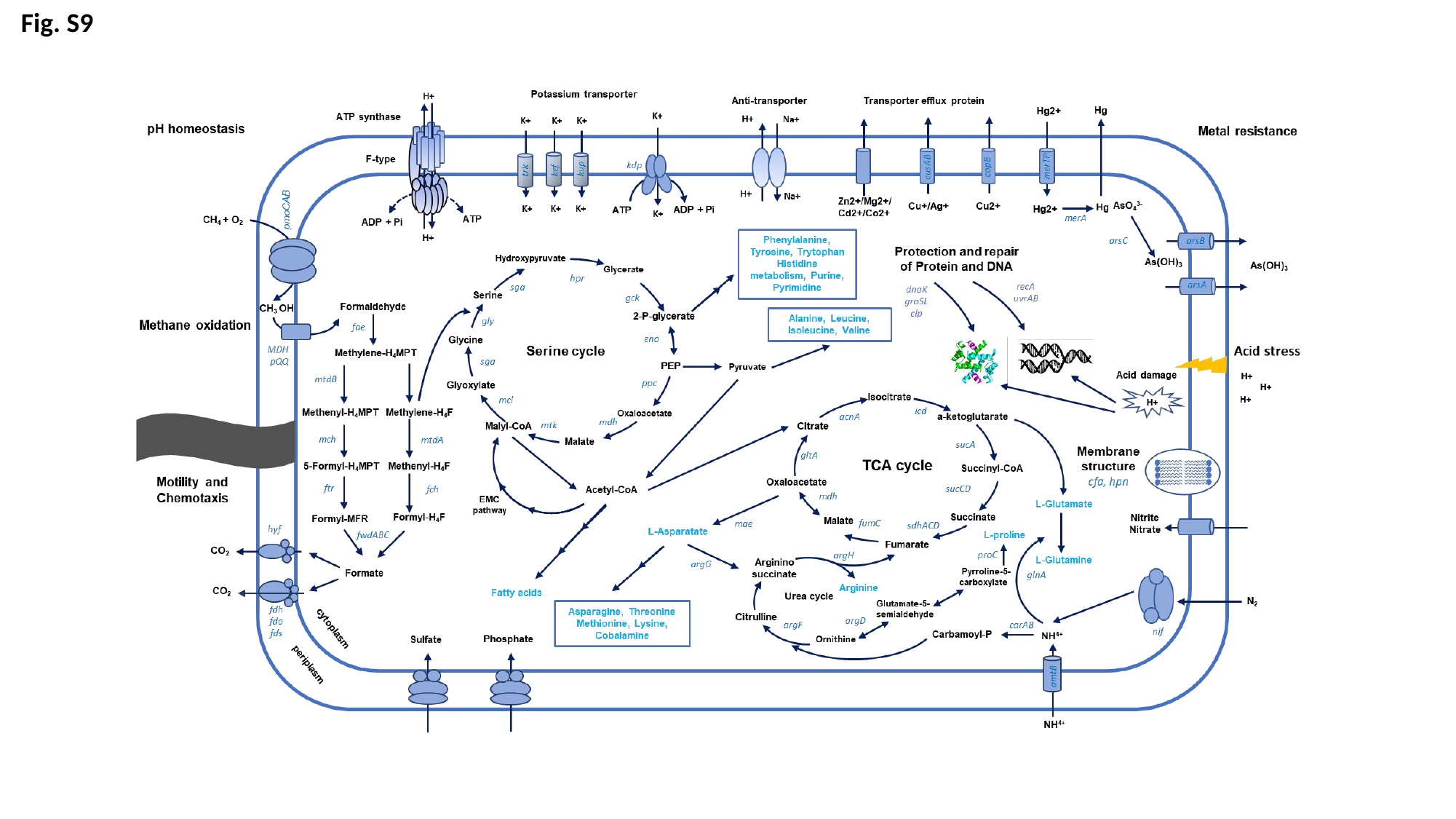

Fig. S9
