# Supplementary material for: Genomic Insights Into the Acid Adaptation of Novel Methanotrophs Enriched From Acidic Forest Soils
Source: Front Microbiol. 2018 Aug 27;9:1982. doi: 10.3389/fmicb.2018.01982 (PMC6119699; doi:10.3389/fmicb.2018.01982)
Supplement: Supplementary file 2 [file Table_1.docx]

***Supplementary Material***

**Genomic insights into the acid adaptation of novel methanotrophs enriched from acidic forest soils**

Ngoc-Loi Nguyen^1#^, Woon-Jong Yu^1#^, Joo-Han Gwak^1^, So-Jeong Kim^2^, Soo-Je Park^3^, Craig W. Herbold^4^, Jong-Geol Kim^1^, Man-Young Jung^4^, and Sung-Keun Rhee^1^*

**#Joint first authors**

***Corresponding author:** E-mail: [rhees@chungbuk.ac.kr](mailto:rhees@chungbuk.ac.kr)

**Supplementary Tables and Figures**

**Supplementary Table S1.** Characteristics of the soils.

| **Soil** | Chungbuk National University | Gutdae Mountain |
| --- | --- | --- |
| GPS | 36°37'33.6"N 127°27'13.3"E | 36°38'43.8"N 127°32'15.4"E |
| Soil type | Forest soil | Forest soil |
| Vegetation | *Pinus koraiensis*  *Quercus mongolica* | *Pinus koraiensis*  *Abies holophylla* |
| pH [1:5]  Cation exchange capacity (mol·Kg^-1^)  Electrical conductivity [1:5] (dS·m^-1^) | 3.89  1.17  0.27 | 4.02  3.81  0.37 |
| Total carbon (%) | 3.80 | 1.55 |
| Total nitrogen (%) | 0.0098 | 0.0164 |
| NH_4_^+^ (mg·kg^-1^) | 19.16 | 10.93 |
| NO_3_^-^ (mg·kg^-1^) | 10.99 | 5.56 |
| Total phosphorous (mg·kg^-1^) | 270 | 385 |
| Salinity (%) | 0.013 | 0.017 |
| Water content (%) | 17.7 | 18.5 |
| Texture | Sandy clay loam | Sandy loam |
| Sand | 58.1 | 68.0 |
| Silt | 17.8 | 13.9 |
| Clay | 24.1 | 18.1 |
| Gene copy/g-soil^-1^ |  |  |
| 16S rRNA | 1.5×10^8^ | 4.4×10^8^ |
| *pmoA* | 5.6×10^6^ | 5.7×10^6^ |

**Supplementary Table S2.** Diversity of the 16S rRNA gene sequences obtained by sequencing PCR amplicons.

|  | R4 | R3 |
| --- | --- | --- |
| Raw reads | 28,606 | 35,540 |
| OTU*^$^ | 139 | 162 |
| Chao1 estimated richness^$^ | 148.4 | 164.7 |
| Shannon’s index for diversity^$^ | 4.438 | 4.391 |
| Simpson’s index for diversity (Inverse)^$^ | 0.9119 | 0.9166 |
| Good’s coverage (%)^$^ | 99.9 | 99.9 |

*An operational taxonomic unit (OTU) was defined as containing sequences with a 97% similarity cut-off.

^$^Alpha diversity metrics were calculated with even sequencing depth (28,606 reads per sample were subsampled).

**Supplementary Table S3.** General features of the methanotrophic and methanotrophic-like contigs, assembled from the enrichment cultures and related strains used in this study. * indicates acidophilic strains.

| **Parameter** | ***Gammaproteobacteria* (*Methylobacter* genus)** | | | | |  | ***Alphaproteobacteria* (*Methylocystis* & *Methyloferula* genera)** | | | | |
| --- | --- | --- | --- | --- | --- | --- | --- | --- | --- | --- | --- |
|  | ***Methylobacter***  **sp. KS41*** | ***M. tundripaludum***  **SV96** | ***M. whittenburyi***  **ACM-3310** | ***M. luteus***  **IMV-B-3098** | ***M. marinus* A45** |  | ***Methylocystis* sp.**  **KS32*** | ***M. bryophila***  **S285 *** | ***M. rosea***  **SV97** | ***M. parvus***  **OBBP** | ***Methyloferula***  ***stellata* AR4*** |
| **Original source** | Acidic soil | Artic wetland soil | Sediment wetlands | Sewage | Marine water |  | Acidic soil | Sphagnum peat | Arctic wetland soil | Soil and fresh water sediments | Acidic forest soils |
| **Accession number** | PHSP00000000 | AEGW00000000 | JQNS00000000 | ATYJ00000000 | ARVS00000000 |  | PHSQ00000000 | NZ_CP019948 | ARCT0000000 | AJTV00000000 | ARWA00000000 |
| **Total bases (Mb)** | 4.74 | 4.85 | 5.44 | 5.03 | 4.99 |  | 3.35 | 4.71 | 3.91 | 4.48 | 4.24 |
| **No. of scaffolds** | 4 | 3 | 7 | 4 | 9 |  | 7 | 1 | 1 | 108 | 1 |
| **CDSs** | 4,226 | 4,608 | 4,949 | 4,595 | 4,523 |  | 3,104 | 4,268 | 3,709 | 4,329 | 3,877 |
| **GC%** | 47.8 | 49.49 | 51.98 | 51.11 | 52.66 |  | 61.27 | 63.15 | 62.5 | 63.35 | 59.54 |
| **CRISPRs** | 3 (4, 91, 63) | 5 (4, 4, 46, 3, 32) | 5 (9, 5, 4, 91, 28) | 1 (12) | 2 (38,26) |  | 0 | 0 | 0 | 6 (4,4, 12,7, 8, 7) | 0 |
| ***pmoCAB* operon** | 1 | 1 | 1 | 1 | 1 |  | 2 | 3 | 2 | 1 | Absent |
| **sMMO-encoding operon** | Absent | Absent | Absent | Absent | Absent |  | Absent | 1 | Absent | Absent | 1 |
| ***pxmABC* operon** | 1 | 1 | 1 | 1 | 1 |  | Absent | 1 | 1 | Absent | Absent |
| **Serine pathway genes** | Incomplete | Incomplete | Incomplete | Incomplete | Incomplete |  | Present | Present | Present | Present | Present |
| **RuMP pathway genes** | Present | Present | Present | Present | Present |  | Absent | Absent | Absent | Absent | Absent |

**Supplementary Table S4.** Central metabolic pathways.

**Supplementary Table S5.** Comparative analysis of the COGs of KS32 and reference species.

**Supplementary Table S6.** Comparative analysis of the COGs of KS34 and reference species.

**Supplementary Table S7.** Genes involved in acid adaptation.

**Supplementary Table S8.** CRISPRs loci and the CRISPR-Cas gene of KS42.

**Supplementary Figure S1.** Methane oxidation activities of the subsamples of the cultures enriched at pH 4 and pH 3. The subsamples of the sequencing batch reactors (SBR) were transferred to a 150-mL gas-tight serum vial with 25 ml of medium containing 10% inoculum. The serum vials were sealed with a butyl rubber stopper, and 20% of pure methane was added to the head space. Methane oxidation was calculated by measuring the disappearance of methane during the incubation.

**Supplementary Figure S2.**  Coverage-coverage plots for two metagenomes illustrating the genome bins of the uncultured methanotrophs, enriched from the acidic soils. The scaffolds of the metagenomes in plot (A) and (B) were from the cultures derived from R4 and R3, respectively. The raw DNA sequence reads were hybrid-assembled into scaffolds and were binned using the sequencing depth of the DNAs. Two dimensional coverages were referred to separate the contigs from different genomes. For example, several genomic bins of the uncultured strains analyzed in this study were marked.

**Supplementary Figure S3.** Phylogenetic tree, based on (A) the 16S rRNA gene and (B) the *PmoA* sequences of KS32 and KS42, showing the phylogenetic relationship for the methanotrophs from the enrichment cultures and related taxa from *Alphaproteobacteria*. The tree was constructed using neighbor-joining methods (Kimura 2-parameter/JTT matrix-based model). Bootstrap values of >50 % (based on 1,000 replicates) from the neighbor-joining and maximum likelihood methods are indicated at branch points. The GenBank accession numbers are shown in parentheses. The bar represents substitutions per divergent position.

**Supplementary Figure S4.** Phylogenetic tree, based on (A) the 16S rRNA gene and (B) the PmoA sequences of KS41, showing the phylogenetic relationship for the methanotrophs from the enrichment cultures and related taxa from *Gammaproteobacteria*. The tree was constructed using neighbor-joining methods (Kimura 2-parameter/ JTT matrix-based model). Bootstrap values of >50 % (based on 1,000 replicates) from the neighbor-joining and maximum likelihood methods are indicated at branch points. The GenBank accession numbers are shown in parentheses. The bar represents substitutions per divergent position.

**Supplementary Figure S5.** Phylogenetic tree of concatenated PmoCAB proteins in gammaproteobacterial methanotrophs. The tree was constructed by maximum likelihood using the Le Gascuel model, based on a concatenation of a total length of 998 amino acids with 1000 bootstraps. Bootstrap values of > 60 % (based on 1,000 replicates) from the maximum likelihood method are indicated at branch points. The scale bar represents 0.05 change per amino acid position. Two type-II methanotrophs, *Methylocapsa acidiphila* B2^T^ and *Methylocystis parvus* OBBP^T^, were used as the outgroup. Evolutionary analyses were conducted with MEGA7.

**Supplementary Figure S6.** Coverage plot of the mapped reads to the scaffolds containing rRNA operons and methane monooxygenase operons of two genome bins: (A) KS32 and (B) KS41. Top track: even coverage of the genome scaffolds. For the mapping of the regions of the scaffolds of KS32 and KS41, total Illumina reads of the metagenomes of the cultures derived from R3 and R4 were used, respectively. Second track: close-up mapped reads to the regions surrounding the rRNA and monooxygenase operons. Bottom track: annotated genes in the scaffolds. Arrows depict the coding strand.

**Supplementary Figure S7.** Phylogenetic tree of concatenated (A) UrtABCDF proteins and (B) KdpFABCD proteins. The tree was constructed with maximum likelihood using the JTT matrix-based model, based on a concatenation of a total length of 1964 and 2741 amino acids, respectively, with 1000 bootstraps. Bootstrap values of > 60 % are indicated at branch points. The scale bar represents 0.01 change per amino acid position.

**Supplementary Figure S8.** (A) Phylogenetic tree of the NtpC/K subunit of the V- or F-type ATP synthase. Numbers on the tree nodes indicate bootstrap values (1000 replicates). The scale bar indicates 0.1% sequence difference. (B) The ATP-binding catalytic sites, according to the crystal structure of subunits A and B of ATP synthase. (C) Sequence alignment of the K subunit of KS41 with those with known protein structures. Among these residues, the conserved H^+^/Na^+^-binding sites are marked by an asterisk, the polar residues flanking the conserved site are in red, the slightly polar residues are in orange, and the hydrophobic residues are in blue. The functional H^+^/Na^+^ selectivity, due to different concentrations of Na^+^ vs. H^+^ in physiological settings, is shown at the right end of the alignment.

**Supplementary Figure S9.** Schematic representation of the metabolic pathways and processes involved in the adaptation of KS32 to acidic conditions.
